# An intelligent ethereum blockchain technology for pest detection and smart irrigation in IoT using hybrid deep learning model

**DOI:** 10.1038/s41598-026-55818-w

**Published:** 2026-06-13

**Authors:** Kiran Bharadwaj Vedula, Rajesh Arunachalam, Sumanth Venugopal

**Affiliations:** 1 Department of Computer Science and Engineering, Saveetha School of Engineering, Saveetha Institute of Medical and Technical Sciences, Thandalam, 602105 Chennai, Tamilnadu India; 2 Department of Electronics and Communication Engineering, Saveetha School of Engineering, Saveetha Institute of Medical and Technical Sciences, Thandalam, 602105 Chennai, Tamilnadu India; 3 Manipal Institute of Technology Bengaluru, Manipal Academy of Higher Education, Manipal, India

**Keywords:** Internet of things, Smart farming system, Ethereum blockchain, Hybrid convolution adaptive recurrent MobileNet, Improved secretary bird optimization, Biological techniques, Plant sciences, Engineering

## Abstract

This research discusses the incorporation of IoT with blockchain technique to enhance the efficiency of smart farming systems, particularly focusing on plant disease classification, pest detection, and smart irrigation. The study aims to develop a secure and effective IoT-based smart farming framework using the Ethereum blockchain to store and transmit data, and a Hybrid Convolution Adaptive Recurrent MobileNet (HC-ARMNet) model for predictive analytics, optimized by the Improved Secretary Bird Optimization (ISBO) algorithm. The research employs IoT sensors to acquire real-time data, which is then stored in the Ethereum blockchain to ensure security. The HC-ARMNet model, combining 1D/2D convolutions with recurrent connections, processes this data for pest detection and irrigation management. The ISBO algorithm is leveraged to fine-tune the technique’s parameters. Datasets used: The proposed system utilizes three standard datasets for evaluation. The PlantifyDr Dataset is used for classifying plant disease, and the Pest Detection Dataset is used for recognizing pests. Also, for the smart irrigation process, the significant field images are collected manually. The accuracy, precision, and FNR rates of the ISBO-HC-ARMNet-aided plant disease classification are 94.16%, 94.2% and 5.87%. At the same time, the ISBO-HC-ARMNet-based pest detection process’s accuracy, sensitivity, and specificity are 93.78%, 93.79% and 93.76%, respectively. In addition, the ISBO-HC-ARMNet-based smart irrigation task’s MSE is 3.21, SMAPE is 0.03, and MASE is 30.23. Thus, the designed system showcases promising performance over classical approaches in terms of accuracy and error rates for plant disease classification, pest detection, and smart irrigation. The research concludes that the IoT-aided smart farming framework with blockchain and the HC-ARMNet model provides a robust solution for secure and efficient agricultural management. The system’s predictive capabilities provide accurate and timely data analysis, facilitating to the improvement of precision agriculture. Future work will focus on improving the system with advanced feature extraction strategies to reduce processing time.

## Introduction

Smart agriculture has recently focused on improving the agriculture domain with the infrastructure to utilize modern technology, including machine learning, remote sensing, autonomous vehicles, robotics, satellite technology, big data, cloud computing, and the IoT for locating, monitoring, automating, and evaluating the farm activities and blockchain to connect the producers with the market^1^. Due to these techniques, real-time data in terms of weather conditions, crop diseases, crop growth, soil conditions, and other associated activities, such as the supply chain for fruits and vegetables, and the data on food safety can be collected and validated. This model is executed by the IoT that gives real-time insights into the weather conditions, soil moisture levels, and crop health, allowing the farmers to make informed decisions and refine their tasks^2^. This data can be evaluated to make the well-known options regarding fertilization, irrigation, and other crop management aspects, leading to very effective and sustainable farming activities^3^. The smart agriculture model plays an important role in supporting farmers to minimize waste, optimize crop yield, and improve sustainability. The IoT has an important impact on numerous human lives, both in professional and personal sectors^4^. It utilizes machines to manage the tiresome work, automate the mundane tasks, and greatly improve the human’s well-being by improving a healthier, much comfortable, and profitable lifestyle. One organization highly influenced by the IoT is agriculture, which has shown improvements in the effective and precise crop management and farms via the utilization of automation, data analytics, and sensors^5^.

The blockchain guarantees the data collected from IoT systems remains transparent and tamper-proof. Each section of data is effectively stored and cannot be changed retroactively, improving the security and trust in the farming task^6^. The blockchain allows the farmers to locate each phase of the farming task, from the seed to harvest, and more. This transparency is significant for quality control, food safety, and compliance with the standards. The IoT systems, such as sensors, can observe crop health, humidity, temperature, and soil moisture in real-time^7^. Combining this data with the blockchain allows better resource management, such as accurate fertilization and irrigation, thus results in better yields and minimizing resource wastage. It allows the agricultural product’s authentication by storing its overall journey on the immutable ledger^8^. This supports minimizing the drawbacks and confirms that users can trust the quality and origin of the products they purchase^9^. By decentralizing data management and storage, the blockchain mechanism supports the farmers by providing them with better control over their data and minimizing their dependence on third parties^10^. This data democratization can result in fairer and highly inclusive agricultural devices.

Some of the real-time criteria utilizing the machine learning mechanisms with sensors allow innovations, including household, mobile phones, military, medical, and transportation appliances^11^. In recent times, some environmental changes have affected the field and the crop conditions, and IoT-aided devices^12^ support farmers in improving production and minimizing the expenses of yield^13^. Present wireless communication models are combined with the cloud sectors to assist the development of smart agriculture and may improve the quality of the product and the production productivity^14^. But, the agriculture-aided tasks may be accurately achieved by employing a more sustainable and reliable manner concerning the feedback, monitoring, transmission, identification, and sensing capabilities^15^. The secured technologies highly conduct authentic tasks in a distributed manner and achieve network integrity^16^. The private data of agriculture should be trusted and secured from unauthorized access until it is obtained on accepted processing and storage systems. The security systems for the IoT devices not only provide robust data on the farmer systems but also minimize the threats to sustainable transmission^17^. Hence, a hybrid technique for the agricultural network employing deep learning prevents the extra overhead on the IoT systems. In addition, the implemented system encourages the security model with numerous mechanisms and secures the IoT data from difficult situations.

### Novelty of the designed framework

The novelty of the suggested research work lies in the integrated algorithmic and architectural synergy among the Ethereum blockchain, IoT, and the newly developed HC-ARMNet technique optimized by the ISBO algorithm. Though the existing studies have leveraged MobileNet or existing CNNs for disease or pest detection, this designed framework presents a hybrid 1D/2D convolutional structure with adaptive recurrent layers, enabling simultaneous extraction of spatial-temporal features from the heterogeneous IoT image and sensor data. This hybridization improves the contextual understanding of the approach, resulting in highly accurate irrigation and pest predictions. Moreover, unlike the existing IoT-blockchain integrations that depend on basic consensus approaches or centralized cloud processing models, this work utilizes Ethereum-based decentralized smart contracts for tamper-proof, secure, and transparent data transmission and storage, guaranteeing end-to-end trust across the agricultural network. The utilization of the ISBO algorithm for hyperparameter optimization further contributes to novelty by enhancing predictive stability and model convergence under dynamic field conditions. This integration of blockchain-enabled data integrity and adaptive deep hybrid modeling specifies a specific advancement over existing models, providing a scalable, secure, and intelligent model tailored for precision agriculture applications.

### Justification for the HC-ARMNet model with its components

The integration of hybrid 1D/2D convolutions, recurrent layers, and MobileNet in the suggested HC-ARMNet method is developed to manage the distinct and complex behavior of the IoT-based agricultural data rather than being over-engineered. The 1D convolution effectively executes the sensor data, whereas the 2D convolutions retrieve the spatial features from the image data associated with the pest detection and plant disease. The inclusion of MobileNet guarantees a computationally efficient and lightweight model suitable for deployment on IoT edge systems. The recurrent layers capture the dynamic environmental variations and temporal dependencies crucial for accurate pest monitoring and irrigation prediction. Optimizing this hybrid model by employing the ISBO further improves the convergence and minimizes the training complexity. Hence, each component plays a complementary and different role in increasing the efficiency, accuracy, and adaptability across multimodal agricultural data streams within the Ethereum-secured IoT system.

The designed IoT-aided smart farming framework, using the blockchain mechanism consists of below contributions.

- To construct a novel IoT-aided smart farming framework employing a blockchain mechanism that classifies the plant disease, recognises the pests, and performs the smart irrigation process securely and accurately. This minimizes the farmer’s workload and time and eliminates the security concerns.
- To suggest blockchain technology for ensuring precision and security in smart farming systems. Moreover, by utilizing the smart contract, the node authentication is performed.
- To develop an HC-ARMNet technique by integrating the recurrent MobileNet and a hybrid convolution with an adaptive concept that improves the precision of the disease classification, pest detection, and smart irrigation process. Here, the ISBO is utilized for tuning the recurrent MobileNet technique’s parameters.
- To suggest a new ISBO algorithm by modifying the traditional SBO with a newly derived strategy that highly improves the convergence rates and decreases the computational time.
- To analyze the effectiveness of the implemented IoT-aided smart farming framework by utilizing numerous performance measures and comparing the existing methods and algorithms.

The structure of the implemented IoT-aided smart farming system using blockchain is presented as follows. Section "Existing works" provides further explanation on the traditional IoT-based smart farming works. Section "Novel framework of Data storage in ethereum blockchain technology and pest detection" explains the novel framework of data storage in Ethereum blockchain technology and pest detection. Section "Modified secretary bird optimization method for parameter optimization and HC-ARMNet" elucidates the ISBO for parameter optimization and HC-ARMNet. Section "Plant disease classification, pest detection, and smart irrigation using developed HC-ARMNet" defines disease classification, pest detection, and smart irrigation using the developed HC-ARMNet. Section "Results and discussions" showcases the experimental examination of the presented IoT-aided smart farming system. Finally, Section "Conclusion" concludes the implemented IoT-driven smart farming process.

## Existing works

### Related works

In 2024, Ahmed *et al.*^18^ have implemented a process named Cluster Head Sleep Schedule (CHSS), which has an effective data collection mechanism for smart agriculture. The objective was to improve the task of data acquisition within a high-scale agricultural infrastructure, where numerous sensors simultaneously produce enormous data while observing and securing the crops from pest attacks. This mechanism was developed to minimize data redundancy and support immediate pest attack control.

In 2022, Kumar *et al.*^19^ have recommended a smart contract and deep learning to suggest a new IoT-aided method for allowing secure data communication between its numerous applications. In particular, new key management and authentication approaches were suggested to guarantee secure data transmission. The smart contract-aided consensus strategy was utilized on valid transactions that ensured and included the generated blocks in the blockchain. A detailed comparative experiment was conducted over conventional strategies.

In 2021, Awan *et al.*^20^ have presented a smart mechanism with a new strategy for the conventional agricultural transformation to smart agriculture, considering both IoT and blockchain properties. The model provided automatic, reliable, and open food tracks constructed with the properties of blockchain IoT systems. For experimental purposes, the designed approach was contrasted with other methods.

In 2023, Saba *et al.*^21^ have presented a smart optimization system to implement a quality-aware, sustainable, and reliable agriculture employing machine learning. Initially, the implemented system employed smart systems to automate data aggregation and communication. Next, this system validated the trust-aided decentralized blockchain-aided security features for combining the trusted model to minimize the interference of the communication. Various studies have been conducted and have illustrated their effectiveness.

In 2023, Hossain *et al.*^22^ have explored a high-potential IoT machine learning-employed farm management module. By employing cutting-edge innovations such as machine learning and IoT, the designed system improved the traditional farm management models. The presented work also employed the blockchain to generate a decentralized and safe network that ensured the integrity of the data and reduced resource utilization. The presented work provided more services to the farmers.

In 2022, Ahmed *et al.*^23^ have presented a sustainable and smart infrastructure that employed Artificial Intelligence (AI), IoT systems, and cloud computing to perform and achieve the requisite data. The model applied digital analytics and secured the outcomes in decentralized cloud data sources via blockchain to improve numerous applications. In addition, the layer-aided architecture enabled a reliable incentive framework that could support protected and secured smart city applications.

In 2021, Praveen *et al.*^24^ have recommended a blockchain mechanism in the domain of agriculture that could produce significant outcomes by generating direct markets for the farmers, as the components of blockchain were capable of locating the origin of appetite. In this, the Regression Technique (RT) was used to evaluate the data acquired from the IoT sensors to identify and handle plant diseases effectively. The model was examined in numerous applications and provided very reliable outcomes.

In 2024, Mahalingam *et al.*^25^ have introduced a hybrid Recurrent Neural Elliptical Curve Blockchain (RNECB) to store agricultural data securely in the cloud platform. The developed system filtered the data source by the pre-processing step and utilized in the field monitoring stage. The crypto evaluation was conducted, and the encrypted data was recorded in the cloud platform. The comparative validation ensured that the implemented system provided better solutions than others.

### Research gaps and challenges

Smart farming using IoT-based blockchain mechanism is implemented for secure and efficient management of agricultural processes. IoT devices collect real-time data, such as weather patterns, equipment performance, soil conditions, and crop health, and then securely transfer it through a blockchain network. This method ensures the security of data sharing and increases trust in the supply chain. Yet, IoT-based smart farming using blockchain technologies faces some challenges, which are listed in the points below.

- The conventional IoT-based techniques involve complex technical configuration, and managing the huge amount of data derived from IoT sensors is challenging in terms of storage and processing.
- Securing IoT systems and communication networks against cyber attacks is very difficult. Guaranteeing the security and privacy of agricultural data stored on the blockchain is significant to eliminate malicious access to important data.
- Implementing and maintaining existing IoT-based techniques is expensive, most importantly for small-scale farmers.
- Data quality and reliability are the challenging factors in remote monitoring systems and real-time data transmission.
- Climate change and erratic weather patterns pose significant risks to agriculture. Technologies must be adaptable based on changing environmental conditions and resilient to extreme weather events.

The advantages and demerits of conventional IoT-aided smart farming approaches using blockchain are described in Table 1.

**Table 1:** Advantages and demerits of existing IoT-aided smart farming approaches using blockchain.

Author [citation]	Methodology	Merits	Challenges
Ahmed *et al.*^18^	CHSS	• This technique helps conserve energy and extends battery life, which reduces the overall power consumption.	• This sleep schedule method involves higher initial costs for hardware and software integration.
Kumar *et al*^19^.	CS	• This method ensures secure data transmission within the IoT-enabled agriculture framework that safeguards data integrity.	• The scalability of the blockchain network is still challenging.
Awan*et al*^20^.	IoT	• This centralized system improves overall productivity and helps the farmers by providing a single platform to record and access all relevant details.	• Protecting data security and privacy is challenging.
Saba *et al*^21^.	Trust-based decentralized blockchain	• This method maintains the integrity of agricultural data and prevents unauthorized access.	• The computational burden of this system is more and results in increased energy consumption.
Hossain*et al*^22^.	IoT-ML	• This method can process huge amounts of data rapidly and effectively.	• It needs a vast amount of data for training to obtain optimal efficiency.
Ahmed *et al*^23^.	AI	• This model boosts the speed of connections and links among IoT systems, upgrading the potential for smart applications.	• It is costly due to the high requirements for computation, bandwidth, and potential delays.
Praveen *et al.*^24^	RT	• This method accurately predicts crop yields, minimizing labor expenses and increasing operational efficiency.	• It is not adaptable to varying environmental conditions, which leads to inaccurate predictions.
Mahalingam*etal*^25^.	RNECB	• This method provides strong security and is more scalable.	• This method leads to overfitting issues.

## Novel framework of data storage in ethereum blockchain technology and pest detection

### Proposed framework of ethereum blockchain and iot-aided pest detection

**Fig. 1:**
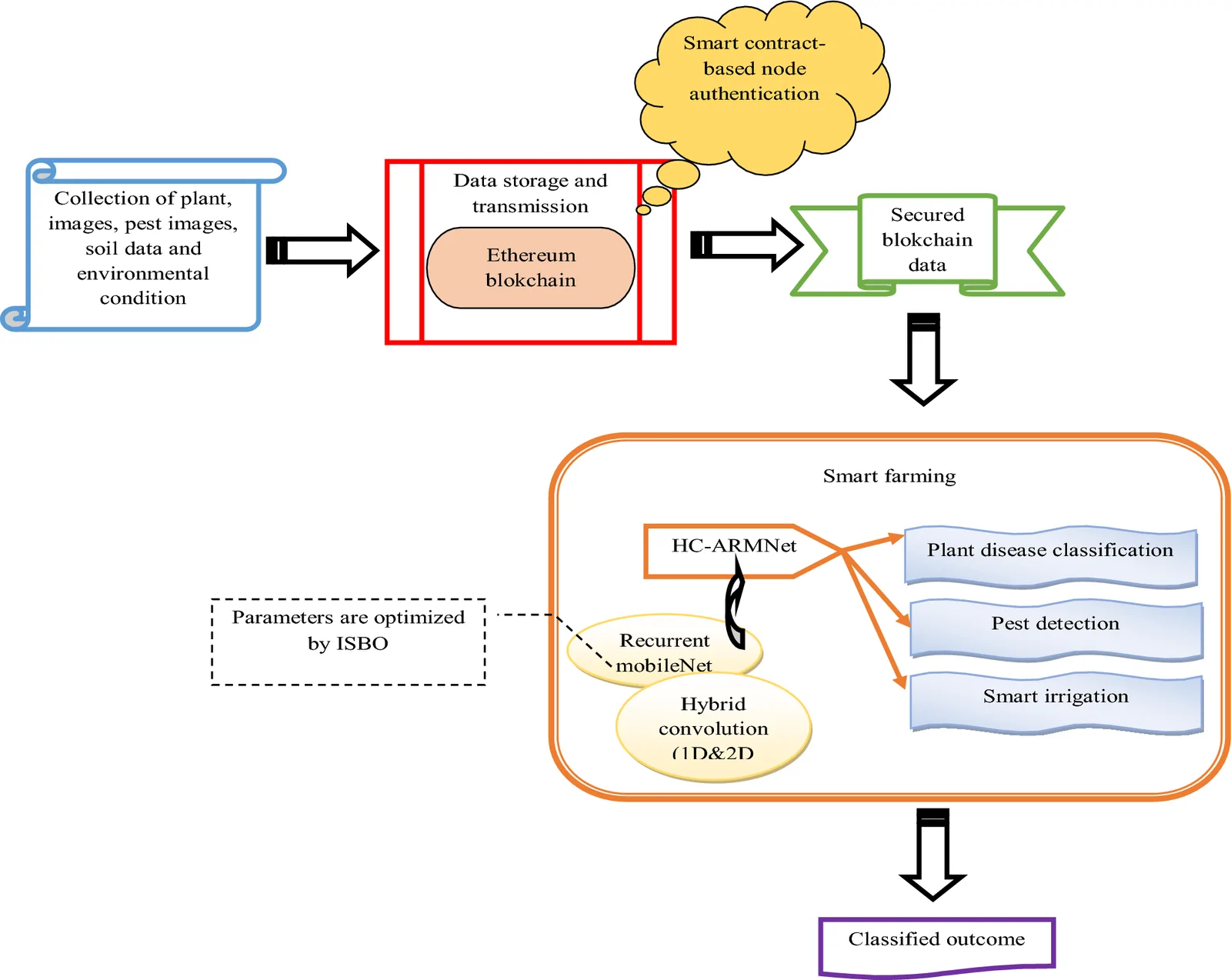
Architectural representation of the implemented IoT-aided smart farming framework using Blockchain.

The IoT has a high impact on numerous aspects. The smart agriculture employed by the IoT^39^ provides real-time context into the weather conditions, soil moisture levels, and health conditions of the crop. This data is validated to support effective decision-making concerning fertilization, irrigation, and other concepts of crop management, leading to highly sustainable and effective farming activities. But the vast amount of data produced from the smart agriculture models can result in complexities in terms of data processing, transmission, and storage. Agriculture is a crucial sector providing a significant food source for providing economic growth and human consumption. This mechanism utilizes technologies such as edge computing, Artificial Intelligence (AI), big data, and cloud computing with the goal of improving agricultural tasks. But smart agriculture’s smart aspects require attention. The involvement of blockchainin the smart agriculture domain provides various advantages to the sector. The blockchain^40^ provides a decentralized and secure ledger device capable of constructing trust between the stakeholders and fostering transparency. Moreover, the involvement of blockchain-aided smart contracts organizes the settlement and payment tasks, minimizing the risk of fraudulent activities and improving overall effectiveness. However, some limitations and vulnerabilities demand ongoing technology’s attention to confirm that the blockchain remains a privacy-preserving and secure technology for smart agriculture applications. Hence, a new effective IoT-aided smart farming system is designed in this work. Figure 1 illustrates the architectural representation of a developed IoT-aided smart farming framework using Blockchain.

An intelligent IoT-aided smart farming system is constructed by utilizing the blockchain mechanism. The IoT sensors gather the soil sensor data and the crop images in real-time and further integrate this data with the blockchain for performing the smart irrigation process. In this, the gathered data is stored and transmitted in the Ethereum blockchain with the hash function to secure the data from suspicious activities. The blockchain mechanism guarantees the precision and security of the data in the smart farming systems. By harnessing real-time information generated by IoT sensors, the HC-ARMN technique is developed to recognize the pests and classify the plant diseases in the agricultural farm. Moreover, this technique supports performing the smart irrigation process. This predictive analytics capacity allows the farmers to make plans for their efficient farming with better capacity. The sustainability in agricultural activities is improved by tuning the parameters in the suggested recurrent MobileNet technique by the ISBO approach. The performance of the introduced IoT-aided smart farming framework employing a blockchain mechanism is contrasted and analyzed with numerous traditional prediction techniques to ensure its success rate.

### IoT-based data collection

IoT is utilized in the agriculture sector to gather data regarding crop growth, environmental conditions, and more. In IoT-aided smart farming, the device monitors the crop field employing the sensors, including moisture of the soil, humidity, temperature, and light, and automates the irrigation process. Here, the IoT sensors are designed to obtain and validate the data from numerous sources to support the farmers in forecasting future conditions. This IoT-based data collection improves productivity by automating the sensor data collection, preventing the requirement for manual collection.

The developed IoT-aided smart farming model includes plant disease classification, pest detection, and smart irrigation tasks. For these operations, the following data sources are employed.

#### PlantifyDr dataset

To classify the plant disease^37^, this dataset is utilized, which includes more than 1,25,000 images in a JPG format. From the overall images, 93,750 images (75%) are supported for training, and 31,250 (25%) images are supported for testing. There are 10 distinct plant types, such as Apple, Tomato, Cherry, Strawberry, Corn, Potato, Peach, Grape, Citrus, Bell pepper and are included in this dataset, and 37 plant diseases are considered in the data source. In this dataset, the data augmentation is already applied.

#### Pest detection dataset

To recognize the pests in the agricultural field^38^, the necessary images are gathered by this dataset. In this dataset, 75,222 images are included, where 75% of images (56,416) and 25% of images (18,806) are utilized for training and testing. It includes 102 classes. Some of the classes are rice leaf caterpillar, rice gall midge, Asiatic rice borer, rice leaf roller, and so on.

#### Smart irrigation dataset

For executing the smart irrigation operations, the necessary field images are gathered manually. There are 2000 field images gathered for this process, where 1500 images (75%) are utilized for model training and 500 images (25%) are utilized for testing. The resolution of the gathered images is 1920 × 1080 pixels, and the classes of this dataset are No irrigation, Moderate and High irrigation.

**Fig. 2:**
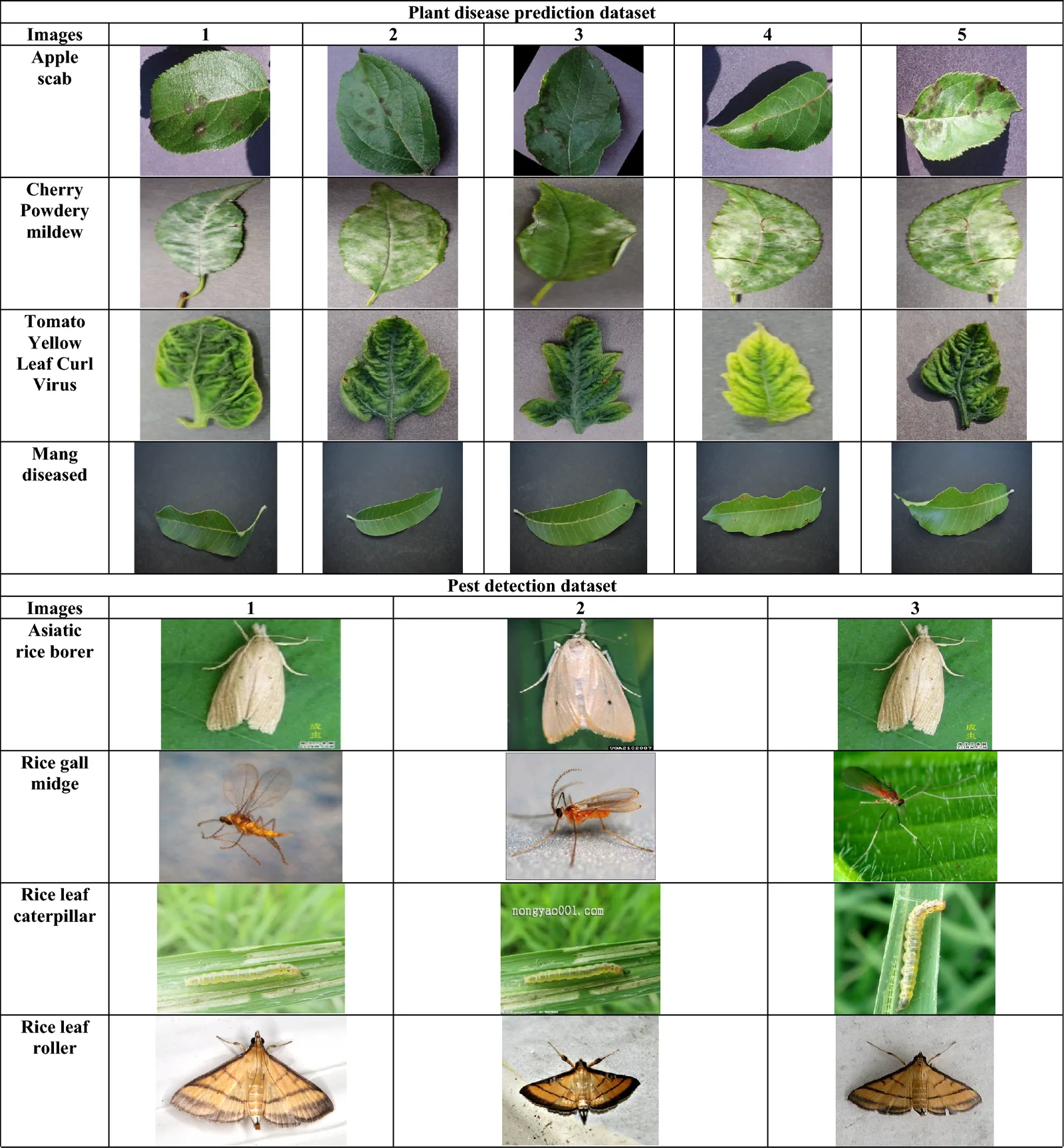

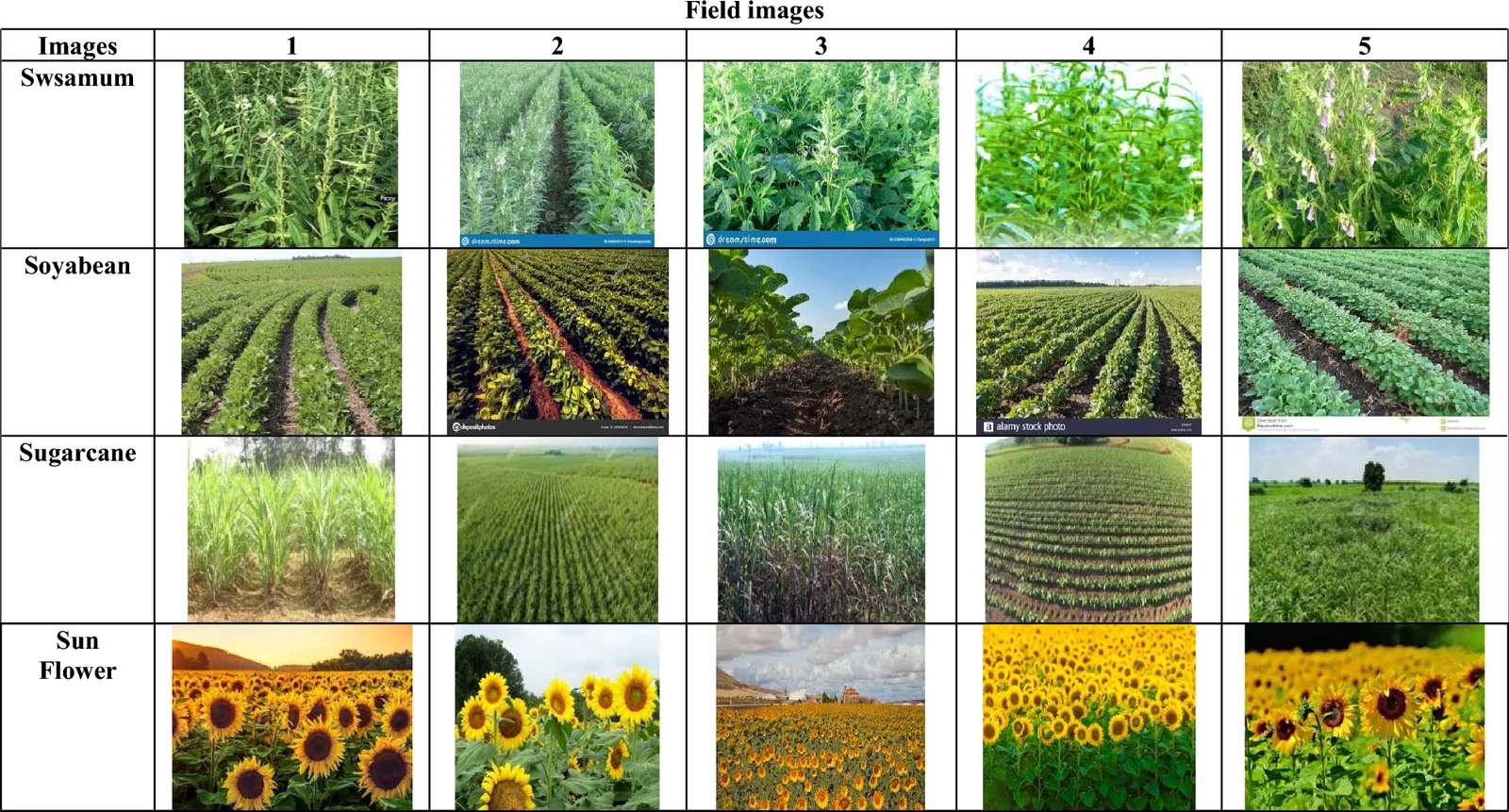
Sample plamt images of the developed IoT-based smart farming system.

From these three datasets, the obtained plant images, field images, and the soil and environment condition data are specified as $Pl_{i} ,\,\,\,here\,\,i = 1,2,...I$,$Fi_{j} ,\,\,\,here\,\,j = 1,2,...J$, and $SE_{d} ,\,\,\,here\,\,d = 1,2,...D$, respectively. Here, the factors $I,J,\,\,\,and\,\,\,\,D$ indicate the amount of gathered plant images, field images, and data, respectively. The gathered sample plant and field images for the implemented IoT-based smart farming system are provided in Fig. 2.

### Data stored and transferred in the ethereum blockchain

**Fig. 3:**
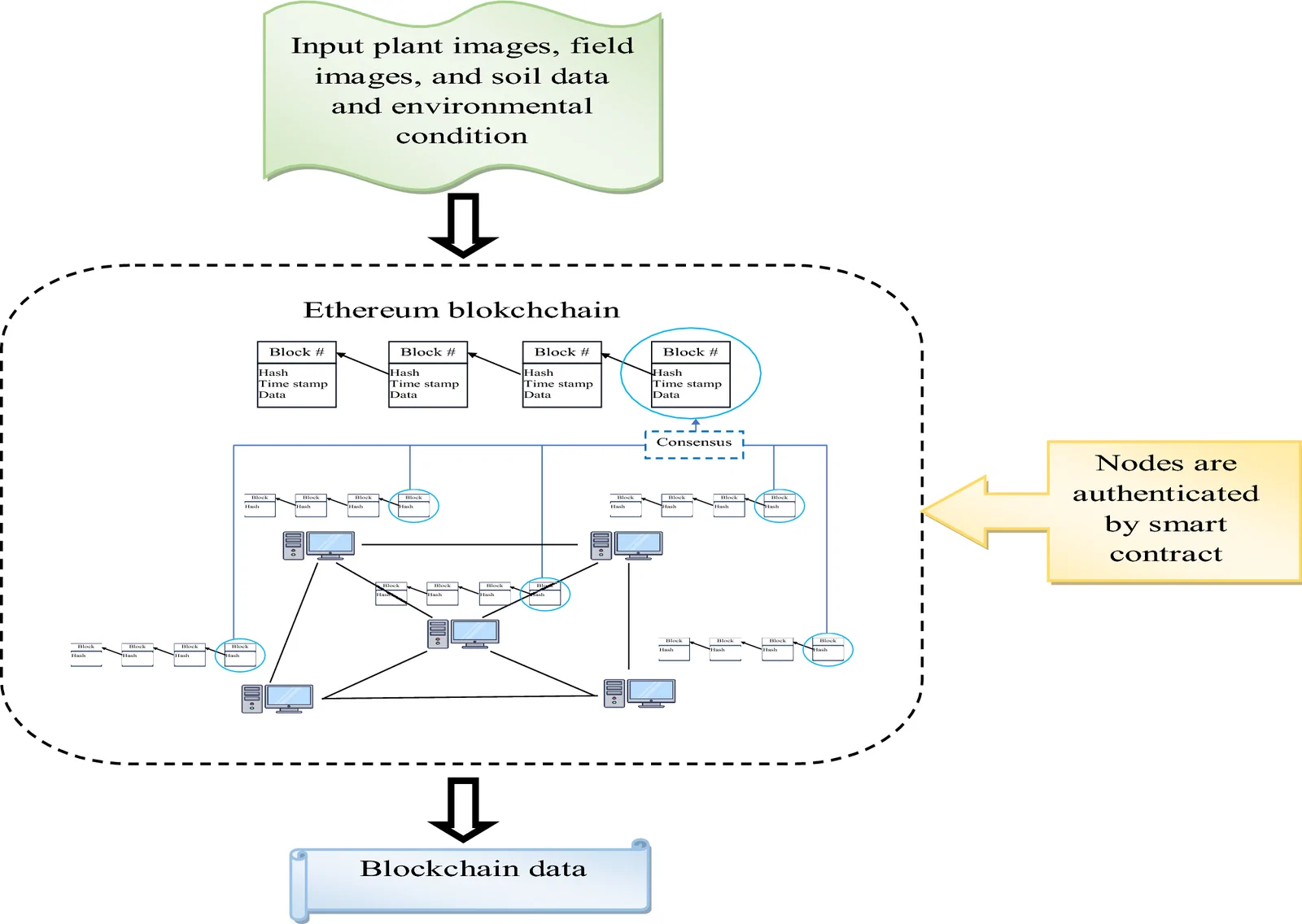
Illustration of Ethereum blockchain-based data storage and transmission.

The blockchain mechanism employs distributed software and computing to transmit the data that can be employed in the agricultural field to strengthen the effectiveness and safety of the supply chain. Ethereum^27^ is the blockchain’s second generation that is employed to construct smart contracts, which are combined with blockchain-aided agriculture devices. Ethereum is a public blockchain that is distributed and open-sourced. It performs as an operating system, where the games and the decentralized applications are implemented. It is focusing on to modify the way the Internet is processed. The Ethereum transactions are managed by the smart contract, not by any person, institution, or government. The Ether is Ethereum’s cryptocurrency. This is employed in financial transactions as digital assets and digital wallets. The utilization of the Ethereum blockchain provides two significant advantages. One is data security. Here, the data storage is secured in the overall supply chain. The second advantage is supply chain management. It improves the transparency and efficiency of the product movement. Moreover, the Ethereum blockchain encourages secure payment tasks with lower transaction costs. Therefore, in this work, the Ethereum blockchain is utilized for the storage and transmission of agricultural data securely. Initially, the gathered field images $Fi_{j} ,$ plant images $Pl_{i}$, and soil data, and environmental conditions $SE_{d}$ for the implemented IoT-based smart irrigation model are given to the Ethereum blockchain. This securely stores the data in the blocks and transmits the data without any modifications or security concerns. Thus, the secured blockchain data $A_{i}^{Bl}$ is obtained from this process. The Ethereum blockchain-based data storage and transmission is demonstrated in Fig. 3.

### Smart contract to the authenticated nodes

The Ethereum smart contract processes on the public network of a large number of nodes. These nodes distribute the blockchain database that includes the product and crop information. All the nodes can interact with each other. The data that is inputted and saved across all the nodes in the network. The details are evaluated via the consensus strategy before recording in the blockchain. Based on the consensus strategy, most of the nodes should agree on the data before being recorded on the blockchain. It is impossible to access this device since it is hard to take control of the overall system, and it consumes more computational power. The transaction hashes, miner address, timestamp, block size, serial number, and more details are included in the Ethereum blockchain. The mining task produces the new block with some miners with the support of a smart contract. Because of the smart contract, the blockchain nodes are authenticated. Therefore, the immutability and the accuracy of the process are improved.

## Modified secretary bird optimization method for parameter optimization and HC-ARMNet

### Proposed ISBO with adaptive concept

The ISBO is the improved algorithm of traditional SBO.

#### Purpose

The ISBO algorithm is implemented for tuning the attributes of the recurrent MobileNet approach. From this, the parameters are learning rate, epochs, and hidden neurons are optimally tuned in an appropriate range. With this process, the designed IoT-based smart farming system improves its precision.

#### Novelty

The existing SBO is employed for implementing the recommended ISBO. The SBO is highly suitable for choosing the optimal solutions and also has the capacity to eliminate the local optima issue. However, the SBO takes much time for the iteration process since it employs a random variable in the range between 0 and 1. This may affect the efficiency of the process. To rectify this difficulty, the random factor is improved by utilizing the fitness values. The development of this random factor helps to perform the iteration effectively, thus improving the efficiency of the overall process. The new random integer *rdm* is expressed in Eq. (1).

1 $$rdm = \frac{cct}{{\left( {bbt + wwt + cct} \right)}}$$

The best and the current fitness values are specified as $bbt\,\,\,\,and\,\,\,ctt$, correspondingly. The worst fitness is denoted as *wwt*. This newly formulated random attribute is employed in Eq. (15) for the exploitation stage. This minimizes the processing time and helps to detect the optimal outcomes quickly. The SBO algorithm’s derivations are explained below.

By considering the survival characteristics of the secretary birds, the SBO^26^ is implemented. The secretary bird is an American raptor famous for its unique characteristics and different appearance. The secretary bird’s survival features include simultaneous prey hunting and escaping from predators. The modelling of SBO is provided as follows.

*Initialization phase:* The existing SBO comes under the type of population-aware meta-heuristic mechanism. In this, each secretary bird is noted as a population candidate of the approach. Eq. (2) is applied for the random secretary bird’s place initialization.

2 $$O_{n,m} = lo_{m} + x \times \left( {up_{m} - lo_{m} } \right),\,\,n = 1,2,...,I,\,\,m = 1,2,...,J$$

The $n^{th}$ secretary bird’s place is denoted as $O_{n}$. The upper and lower search space areas are given as $up_{m} \,\,\,\,and\,\,\,\,lo_{m}$, accordingly. The arbitrary integer is declared as *x*.

In the SBO, the optimization starts for the candidate solution’s outcome in Eq. (3).

3 $$O = \left[ {\begin{array}{*{20}c} {o_{1,1} } & {o_{1,2} } & \cdots & {o_{1,m} } & \cdots & {o_{1,j} } \\ {o_{2,1} } & {o_{2,2} } & \cdots & {o_{2,m} } & \cdots & {o_{2,j} } \\ \vdots & \vdots & \ddots & \vdots & {\mathinner{\mkern2mu\raise1pt\hbox{.}\mkern2mu \raise4pt\hbox{.}\mkern2mu\raise7pt\hbox{.}\mkern1mu}} & \vdots \\ {o_{n,1} } & {o_{n,2} } & \cdots & {o_{n,m} } & \cdots & {o_{n,j} } \\ \vdots & \vdots & {\mathinner{\mkern2mu\raise1pt\hbox{.}\mkern2mu \raise4pt\hbox{.}\mkern2mu\raise7pt\hbox{.}\mkern1mu}} & \vdots & \ddots & \vdots \\ {o_{I,1} } & {o_{I,2} } & \cdots & {o_{I,m} } & \cdots & {o_{I,j} } \\ \end{array} } \right]_{I \times j}$$

The variable *O* is considered as the secretary birds group, and the attribute $O_{n,m}$ is considered as the value of the $n^{th}$ secretary bird’s $m^{th}$ query factor. The secretary group candidate’s count is noted as *I*, and the dimension factor of the issue is given as *j*.

In SBO, each secretary bird refers to the member outcome to tune the issue. Hence, the objective function is evaluated according to the values recommended by all the birds. Eq. (4) formulates objective function values, which are formed in a vector form.

4 $$L = \left[ {\begin{array}{*{20}c} {L_{1} } \\ \vdots \\ {L_{n} } \\ \vdots \\ {L_{I} } \\ \end{array} } \right]_{I \times 1} = \left[ {\begin{array}{*{20}c} {L\left( {O_{1} } \right)} \\ \vdots \\ {L\left( {O_{n} } \right)} \\ \vdots \\ {L\left( {O_{I} } \right)} \\ \end{array} } \right]_{I \times 1}$$

Here, the objective function vector is pointed as *L*. Thefitness function for the $n^{th}$ secretary bird is given as $L_{n}$.

Two different secretary bird characters are modeled as follows.

#### Secretary bird’s hunting mechanism (Exploration)

This hunting task of the secretary birds during predation on snakes is normally classified into three portions.

*Searching for prey-* In this stage, the secretary birds normally start with a search for powerful prey, specifically the snakes. The position updating in this process is modelled employing Eq. (5) and Eq. (6).

5 $$While\,\,\,q < \frac{1}{3}Q,o_{n,m}^{new\,\,H1} = o_{n,m} + \left( {o_{rdm\_1} - o_{rdn\_2} } \right) \times X_{1}$$

6 $$O_{n} = \left\{ {\begin{array}{*{20}c} {O_{n}^{new\,\,H1} ,} & {if\,\,\,L_{n}^{new\,H1} < L_{n} } \\ {O_{n} ,} & {else} \\ \end{array} } \right.$$

In this, the current iteration is given as *q*, and the maximum iteration count is declared as *Q*. The newly determined position of the $n^{th}$ secretary bird in the first phase is specified as $O_{n}^{new\,\,H1}$. The attributes $o_{rdm\_1} \,\,\,\,and\,\,\,\,\,o_{rdn\_2}$ are the arbitrary candidate solutions in the first phase iteration. The randomly produced dimensional array is given as $X_{1}$ from the range [0, 1]. The factor $o_{n,m}^{new\,\,H1}$ is its $m^{th}$ dimension values, and its associated fitness value is given as $L_{n}^{new\,H1}$.

*Prey consumption-*Further, the secretary bird engages in a different hunting strategy. In this stage, the Brownian motion is utilized by employing Eq. (7) to analyze the secretary bird’s random motion. The prey consumption’s position updating process is given in Eq. (8) and Eq. (9).

7 $$Br = rdm\left( {1,j} \right)$$

8 $$\begin{gathered} While\,\,\,\frac{1}{3}Q < q < \frac{2}{3},o_{n,m}^{new\,\,H2} \hfill \\ \,\,\,\,\,\,\,\,\, = o_{bst} + \exp \left( {\left( \frac{q}{Q} \right)^{4} } \right) \times \left( {Br} \right) - 0.5 \times \left( {o_{bst} - o_{n,m} } \right) \hfill \\ \end{gathered}$$

9 $$O_{n} = \left\{ {\begin{array}{*{20}c} {O_{n}^{new\,\,H1} ,} & {if\,\,\,L_{n}^{new\,H1} < L_{n} } \\ {O_{n} ,} & {else} \\ \end{array} } \right.$$

Here, the variable $rdm\left( {1,j} \right)$ explains the arbitrarily generated multi-dimensional array from the normal distribution, and the present best value is denoted as $o_{bst}$.

*Attacking prey-* When the prey is tired, the bird utilizes the chance and immediately takes action, leveraging its leg muscles to perform an attack. In this phase, the position updating of the secretary bird is given in Eq. (10) and Eq. (11).

10 $$\begin{gathered} While\,\,\,q> < \frac{2}{3}Q,o_{n,m}^{new\,\,H2} \hfill \\ \,\,\,\,\,\,\,\,\, = o_{bst} + \left( {\left( {1 - \frac{q}{Q}} \right) \wedge \left( {2 \times \frac{q}{Q}} \right)} \right) \times o_{n,m} \times Lf \hfill \\ \end{gathered}$$

11 $$O_{n} = \left\{ {\begin{array}{*{20}c} {O_{n}^{new\,\,H1} ,} & {if\,\,\,L_{n}^{new\,H1} < L_{n} } \\ {O_{n} ,} & {else} \\ \end{array} } \right.$$

To enrich the SBO’s accuracy, the weighted Levy flight is employed, and that is estimated in Eq. (12).

12 $$Lf = 0.5 \times Levy\left( j \right)$$

In this, the variable $Levy\left( j \right)$ indicates the Levy flight’s distribution function. It is determined in Eq. (13).

13 $$Levy\left( J \right) = v \times \frac{k \times \sigma }{{\left| s \right|^{{\frac{1}{\eta }}} }}$$

Here, the fixed constant is denoted as *v* and set to 0.001. Further, another fixed constant is given as *η* and is set as 1.5. The arbitrary integers in the region of [0, 1] are given as $k\,\,\,and\,\,\,\,\,s$. The variable *σ* is estimated in Eq. (14).

14 $$\sigma = \left( {\frac{{\Gamma \left( {1 + \eta } \right) \times \sin \left( {\frac{\pi \eta }{2}} \right)}}{{\Gamma \left( {\frac{1 + \eta }{2}} \right) \times \eta \times 2\left( {\frac{\eta - 1}{2}} \right)}}} \right)^{{\frac{1}{\eta }}}$$

Here, the gamma function is indicated as *Γ* and the attribute *η* has a value of 1.5.

#### Secretary bird’s escaping mechanism (Exploitation)

The secretary bird’s primary foes are huge predators, including foxes, jackals, hawks, and eagles, which may compete for food sources or attack them. In the development of SBO, it is considered that one of the two conditions below happens with equal likelihood:

- A_1_: Camouflage by the environment.
- A_2_: Run or fly away.

In the initial phase, when the secretary birds recognize predator’s proximity, these birds immediately search for the appropriate camouflage environment. If unsafe and appropriate camouflage environment is nearby discovered, then the secretary birds will opt for quick running or flight to escape. In conclusion, both mechanisms utilized by the secretary birds is designed utilizing Eq. (15), and this updated condition is derived by employing Eq. (16).

15 $$\begin{gathered} O_{n,m}^{new\,H2} \hfill \\ = \left\{ {\begin{array}{*{20}c} {A_{1} :o_{bst} + \left( {2 \times Br - 1} \right) \times \left( {1 - \frac{q}{Q}} \right) \times o_{n,m} ,} & {if\,\,\,rdm < x_{n} } \\ {A_{2} :o_{n,m} + X_{2} \times \left( {o_{rdm} - Y \times o_{n,m} } \right),} & {else} \\ \end{array} } \right. \hfill \\ \end{gathered}$$

16 $$O_{n} = \left\{ {\begin{array}{*{20}c} {O_{n}^{new\,\,H2} ,} & {if\,\,\,L_{n}^{new\,H2} < L_{n} } \\ {O_{n} ,} & {else} \\ \end{array} } \right.$$

Here, the variable *x = 0.5*, and the factor $X_{2}$ denotes the arbitrary creation of a dimensional array. The improved random variable is indicated as *rdm*. The arbitrary candidate outcome of the present iteration is given as $o_{rdm}$. The arbitrary integer selection of 1 or 2 is noted as *Y* that is estimated in Eq. (17).

17 $$Y = round\left( {1 + rdm\left( {1,1} \right)} \right)$$

**Fig. 4:**
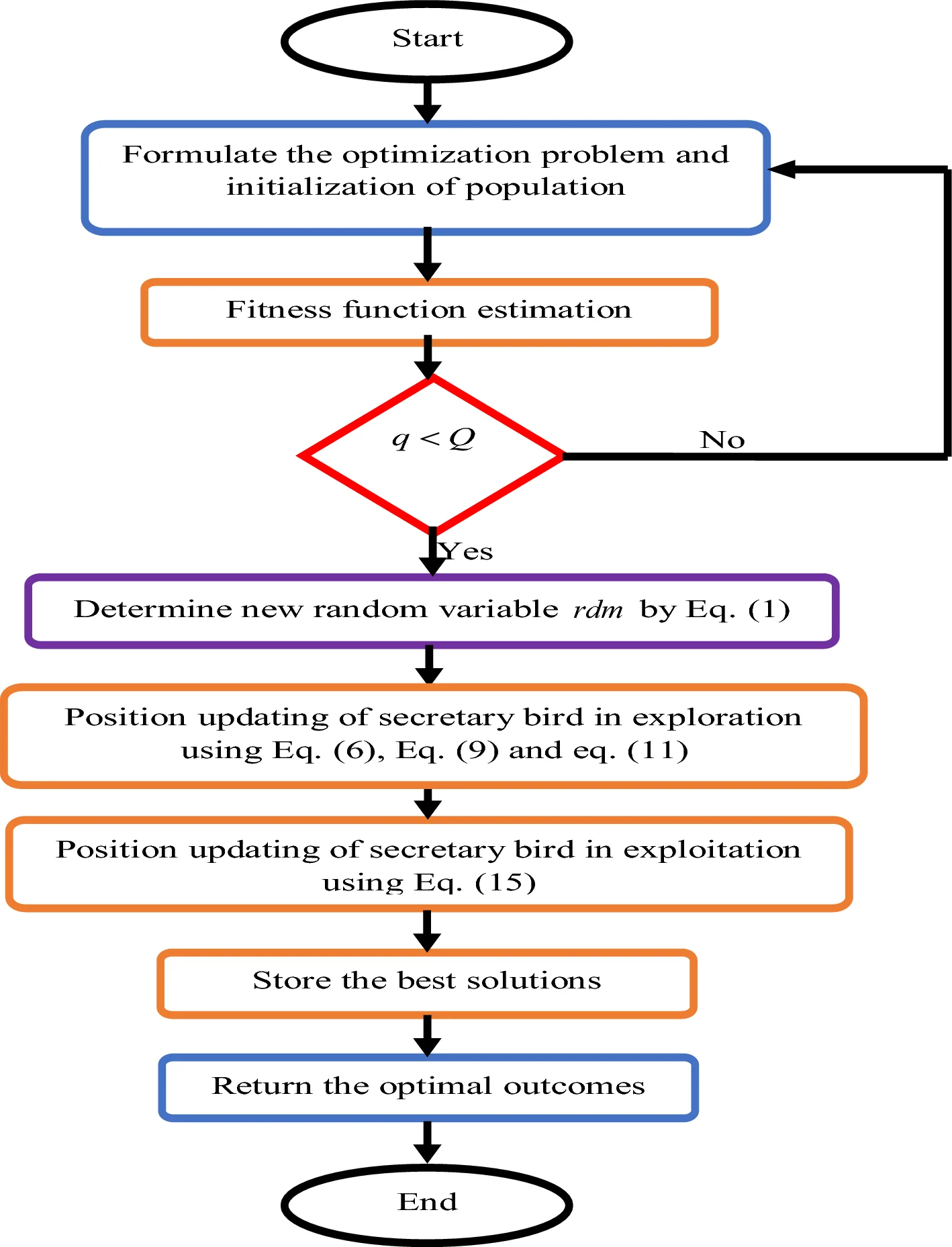
Flowchart of the developed ISBO.

Here, the variable $rdm\left( {1,1} \right)$ specifies an improved arbitrary variable. Algorithm 1 gives the pseudo-code of ISBO, and Fig. 4 shows the flowchart of the developed ISBO.

### Recurrent mobilenet

The recurrent MobileNet is a promising approach for performing classification tasks. To discover highly reliable approaches with lightweight models is the main goal of computer vision and deep learning. Thus, the MobileNet provides very effective solutions. The MobileNet^28^ is a lightweight method and employs depth-wise convolutions to diminish the computational burden while securing promising solutions. The choice of this model is inspired by its suitability and efficacy for processing the images. Because of its lightweight structure, it provides highly accurate and quick solutions. The usage of depth-wise separable convolutions allows the MobileNet to manage a trade-off between classification accuracy and computational requirements. Though the MobileNet produces better solutions, this technique is not suitable for large images. Therefore, the recurrent connections are included in the MobileNet.

**Figure:**
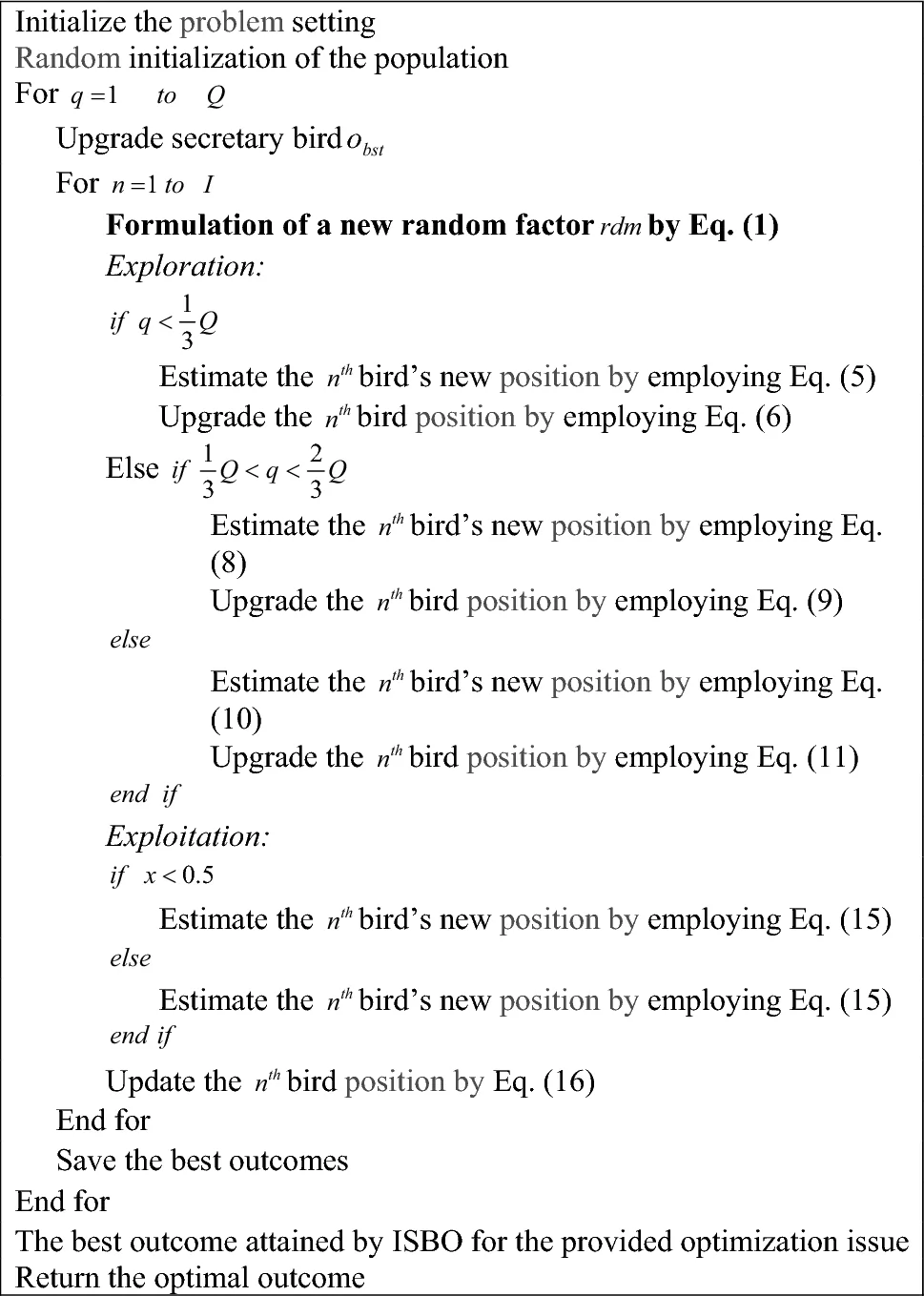
Algorithm 1: Developed ISBO.

The integration of recurrent connections^36^ into the convolution layer in MobileNet forms the recurrent MobileNet. This technique can combine the context data, significant for the classification operations. This mechanism improves the depth of the technique, while the attribute’s count is constant by distributing the weight among the layers. Employing recurrent connections from the result into the hidden layer’s input enables the network to design label dependencies and ease its own outcomes on the basis of its previous outcomes.

### Developed HC-ARMNet for classification and irrigation

The HC-ARMNet technique is implemented for performing plant disease classification, pest detection, and smart irrigation in the suggested IoT-based smart farming mechanism. The HC-ARMNet is the integration of a hybrid (1D/2D) convolution, a recurrent MobileNet with the adaptive mechanism.

#### Handling of 1D sensor data and 2D image data

In the designed approach, the handling of 2D image data and 1D sensor data is carefully developed to preserve the special features of each modality before their fusion in the HC-ARMNet approach. The 1D sensor data, gathered from IoT-based devices such as humidity, soil moisture temperature, and nutrient sensors. This data is passed via a 1D convolutional layer within the HC-ARMNet to retrieve the trend and temporal-based features that reflect the environmental variables affecting the pest activity and pest requirements. On the other hand, the 2D image data is captured by drones or field cameras. Further, these images are processed by 2D convolutional layers of the approach to retrieve the texture and spatial features related to pest existence or the symptoms of plant disease. Once the respective feature representations are achieved, a feature-level fusion approach is employed to combine the temporal dynamics from 1D sensor streams with spatial patterns from 2D images. The integration allows the HC-ARMNet approach to construct a unified representation that supports highly accurate and context-aware pest detection and irrigation control decisions.

The recurrent MobileNet technique has the capacity to produce more accurate solutions. Moreover, this technique demands a very limited amount of computational resources. However, the recurrent MobileNet fails to retrieve the relevant features while processing the vast amount of input images. Hence, the hybrid (1D/2D) convolution is combined into the recurrent MobileNet for getting relevant features even if the input images are high.

The hybrid (1D/2D) convolution^29^ network is employed to recognize the pest by utilizing the crop images and the soil sensor data. Here, the crop images are processed by the 2D convolution, while the sensor data is executed by the 1D convolution. The objective of the hybrid (1D/2D) convolution is to employ multiple dimensional data to extract the high-level features. The deep features are learnt by 1D convolutions, whereas the 2D convolution learns the high-level features. Generally, the 1D convolution includes fully connected and Rectified Linear Unit (ReLU) layers. In addition, the 1D convolution includes max-pooling and four Batch Normalization (BN) layers. The 1D convolution layer’s function is mathematically given in Eq. (18).

18 $$v\left( q \right) = w\left( q \right)*y\left( q \right)$$

In this, the attribute $y\left( q \right)$ is the convolution kernel, and the outcome of the convolution is noted as $v\left( q \right)$. The input of the convolution is specified as $w\left( q \right)$. The initial convolution layer attains a 1D vector and produces the features. The obtained feature is given in Eq. (19).

19 $$w_{n}^{l} \left( q \right) = M\left( {b_{n}^{l} + \sum\limits_{o} {w_{o}^{l - 1} \times y_{no}^{l} } } \right)$$

Here, the variable *M* indicates the ReLU function, and the node bias is indicated as $b_{n}^{l}$. The kernel factors are denoted as $y_{no}^{l}$. The $n^{th}$ node variable is indicated as $w_{n}^{l}$ and the layer is pointed as *l*. For each attribute update, the hidden unit’s distribution is modified. The BN layer’s estimations are given from Eq. (20) to Eq. (23).

20 $$\delta^{l} = \frac{1}{D}\sum\limits_{n} {w_{n}^{l} }$$

21 $$\left( {\alpha^{l} } \right)^{2} = \frac{1}{D}\sum\limits_{n} {\left( {w_{n}^{l} - \delta^{l} } \right)}$$

22 $$w_{n - norm}^{l} = \frac{{w_{n}^{l} - \delta^{l} }}{{\sqrt {\left( {\left( {\alpha^{l} } \right)^{2} + \lambda } \right)} }}$$

23 $$\tilde{w} = \sigma^{l} w_{n - norm}^{l} + \beta^{l}$$

Here, the variables $\beta \,\,\,and\,\,\,\,\sigma$ indicate the arbitrary factors. Then, the attribute *λ* is a small positive integer. The obtained features are fed into the pooling layer. Further, the downsampling task is conducted and formulated in Eq. (24).

24 $$w_{n}^{l} = \mathop {\max }\limits_{\forall r \in \Omega n} \,\,w_{n}^{l - 1}$$

Overall, 64 features are generated by the third and fourth convolution layers. The outcome obtained from these layers is forwarded to the fully connected layer, and it is given in Eq. (25).

25 $$w^{l} = M\left( {b^{l} + w^{l - 1} .y^{;} } \right)$$

Unlike the 1D convolution, there are two max pooling layers present in the 2D convolution. From the first layer, 32 feature maps are generated and from the subsequent layers, 64 feature maps are generated as an outcome. The 1D-2D convolution layers are shown in Eq. (26).

26 $$w^{l} = \left[ {w_{1D}^{l - 1} w_{2D}^{l - 1} } \right]$$

The variables $w_{1D}^{l - 1} \,\,\,\,and\,\,\,\,w_{2D}^{l - 1}$ specify the features learned by the 1D-2D convolution’s fully connected layers. The softmax function and its input are derived in Eq. (27) and Eq. (28).

27 $$w^{m} = \sum\limits_{o} {g_{o} Y_{on} }$$

28 $$stm\left( w \right)_{n} = r_{n} \frac{{\exp \left( {w_{j} } \right)}}{{\sum\nolimits_{o}^{q} {\exp \left( {w_{n} } \right)} }}$$

In this, the weighted connection is specified as $Y_{on}$, and the final layer’s activation function is given as $r_{n}$. Thus, the HC-ARMNet technique is implemented for producing better solutions. This technique can automatically extract the necessary features from a huge amount of inputs, and also, this technique utilizes a very small amount of computational resources. However, the attributes such as epochs, learning rate, and the hidden neurons present in the recurrent MobileNet technique are more, making it difficult to perform the training process. Moreover, these parameters slow down the overall classification process. Therefore, optimally tuning these parameters in the recurrent MobileNet is highly significant. For this objective, the ISBO algorithm is recommended, which is very effective and quickly recognizes the optimal solutions. Hence, an effective HC-ARMNet technique is developed for performing the plant image classification, pest detection, and smart irrigation process in the implemented IoT-driven smart farming strategy. Initially, the blockchain data $A_{i}^{Bl}$ is given to the designed HC-ARMNet technique. The ISBO-based parameter optimization is given in Eq. (29).

29 $$ob = \mathop {\arg \,\,\max }\limits_{{\left\{ {hn^{EB7} ,ep^{EB7} ,lr^{EB7} } \right\}}} \left[ P \right]$$

The hidden neuron in the recurrent MobileNet is denoted as $hn^{EB7}$ and varies from [5–255]. The learning rate in the recurrent MobileNet is indicated as $lr^{EB7}$ and varies from [0.01–0.99]. The epoch count in the recurrent MobileNet is indicated as $ep^{EB7}$ and varies from [5–50]. The precision *P* is maximized in this process and defines the estimation of how close the original and the classified values are to each other. It is formulated in Eq. (30).

30 $$P = \frac{dx}{{dx + jx}}$$

Here, the attributes $dx\,\,\,and\,\,\,jx$ indicate the true positive observations and false positive rates, respectively.

**Fig. 5:**
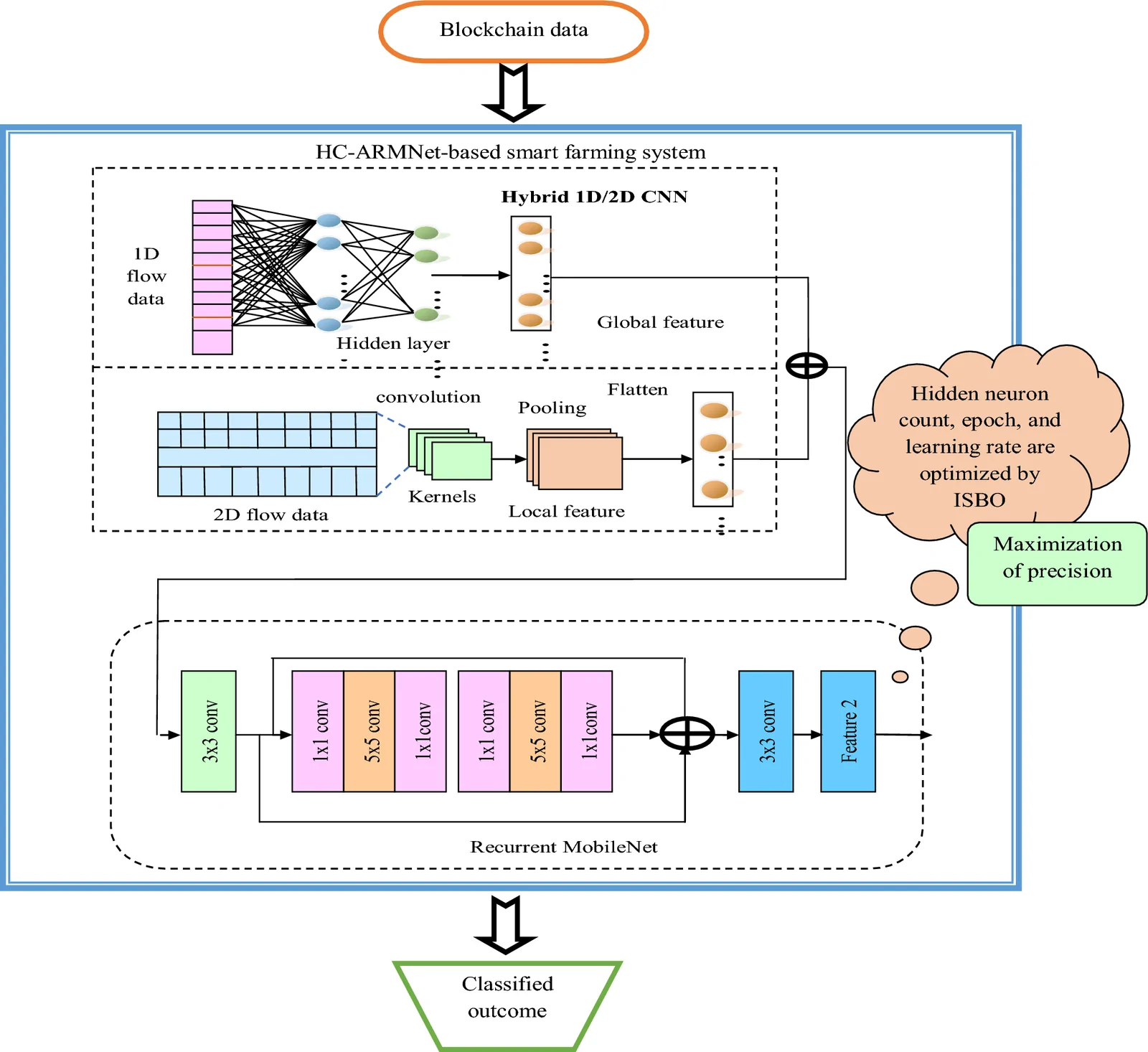
Pictorial representation of the developed HC-ARMNet technique for an IoT-aided smart farming framework.

Thus, the blockchain data $A_{i}^{Bl}$ is processed in the recurrent MobileNet technique and produces highly effective classified outcomes for the developed IoT-aided smart farming framework. The pictorial representation of the HC-ARMNet technique is shown in Fig. 5.

Algorithm 2 gives the pseudo-code of the HC-ARMNet technique.

**Figure:**
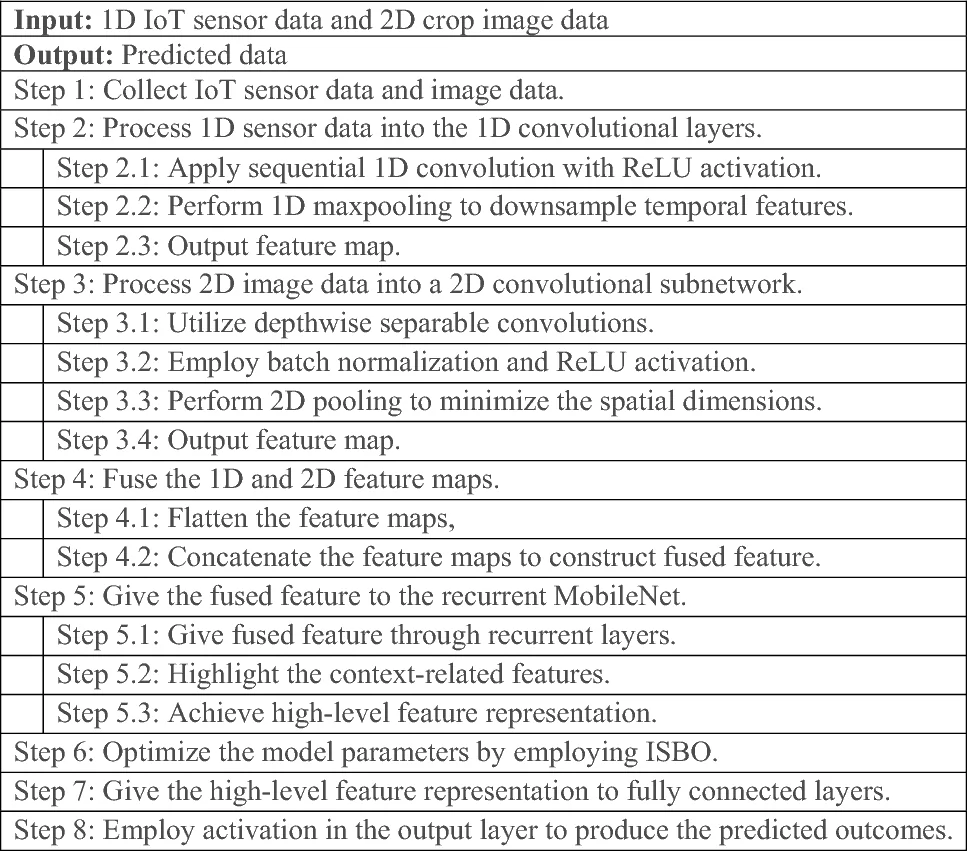
Algorithm 2: Designed HC-ARMNet

## Plant disease classification, pest detection, and smart irrigation using developed HC-ARMNet

### Objective 1: Plant disease classification

**Fig. 6:**
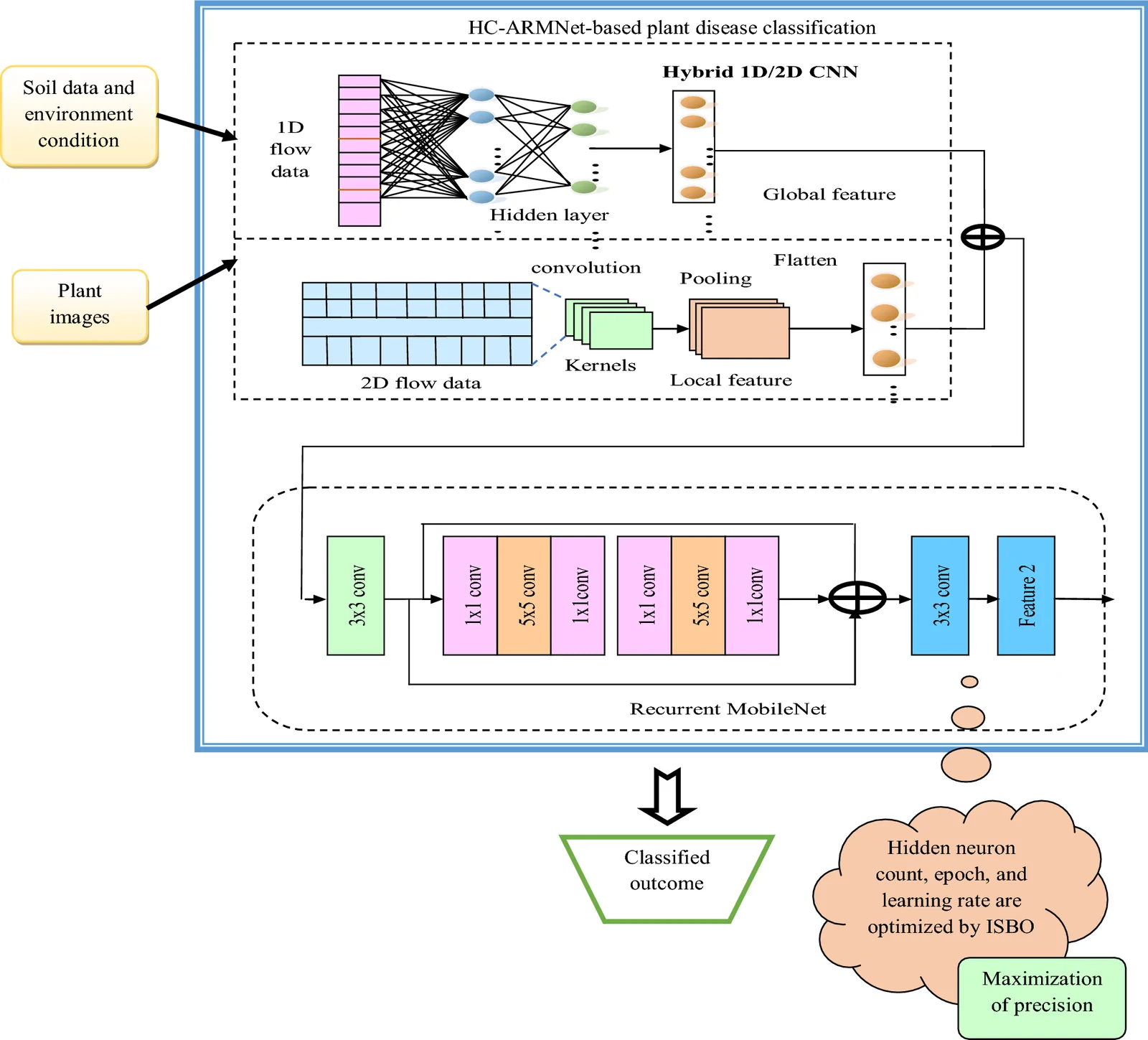
HC-ARMNet-based plant disease classification for IoT-based smart farming system.

The IoT-based smart farming system is presented with the support of a deep learning concept. The developed smart farming system includes three primary objectives. The first objective of the smart farming system is the plant disease classification process. Plant diseases are a threat to the agricultural sector, leading to important financial losses and food security problems. Understanding the disease classifications, symptoms, and features is crucial for long-term crop productivity and efficient disease control. Plant diseases are categorized by three causative agents that are categorized into three types: viral, bacterial,

and fungal disease. Plant diseases are also caused by non-infectious causes, including inheritance problems, stresses from the environment, and nutritional inadequacies. The symptoms of plant disease vary based on the causative agent. Therefore, classifying the plant disease is necessary for minimizing the loss. AI growing in the modern day providing significant disease detection by employing numerous algorithms given into it and employs software for evaluation and maintenance of the disease in the starting stages. Numerous plant disease classification approaches have been presented in previous years utilizing machine learning and deep learning strategies. The machine learning-based plant disease classification techniques have poor interpretability and utilize more processing time. Likewise, the existing deep learning-based strategies generate highly complex networks that minimize their effectiveness. Hence, this work presents an efficient HC-ARMNet technique. Here, from the blockchain data $A_{i}^{Bl}$, the gathered and secured plant images are given to the 2D convolution, whereas the soil and environment condition data are provided to the 1D convolution as input. From the hybrid (1D-2D) convolution, significant features are attained, and these features are executed by the recurrent MobileNet technique. Here, the ISBO algorithm is utilized for optimizing the parameters of the current MobileNet technique. Finally, the plant disease classified outcome is achieved. The HC-ARMNet-aided plant disease classification process is depicted in Fig. 6.

### Objective 2: Pest disease detection

**Fig. 7:**
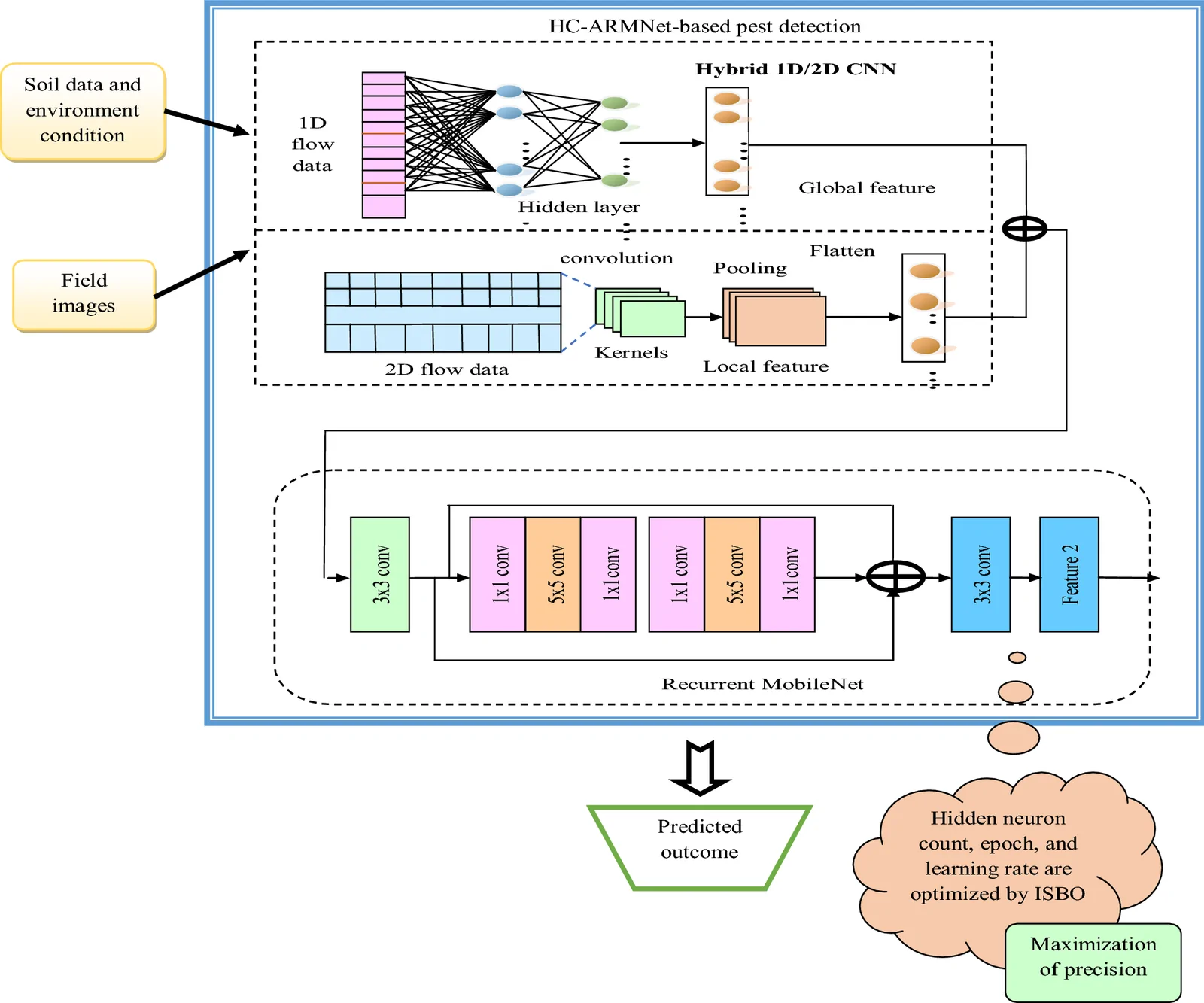
HC-ARMNet-based pest detection technique for the developed IoT-based smart farming system.

Though some traditional deep learning-based techniques offer better outcomes for pest detection techniques, these techniques fail to recognize tiny pests. Moreover, these techniques fail to produce highly accurate solutions. Hence, this work leveraged the HC-ARMNet approach for identifying the pests in the images. This technique is highly effective and capable of recognizing pests even if it is small in size. In this process, from the blockchain data $A_{i}^{Bl}$, secured field images are given to the 2D convolution, and the soil and environment condition data are provided to the 1D convolution as input. From the hybrid convolution process, the significant features are extracted and processed in the recurrent MobileNet for detecting pests. Here, the parameters of the recurrent MobileNet technique are optimized by the designed ISBO. Thus, the pest-detected outcome is achieved in the end with the support of the HC-ARMNet technique. The structural illustration of the HC-ARMNet-based pest disease detection is displayed in Fig. 7.

### Objective 3: Smart irrigation system

**Fig. 8:**
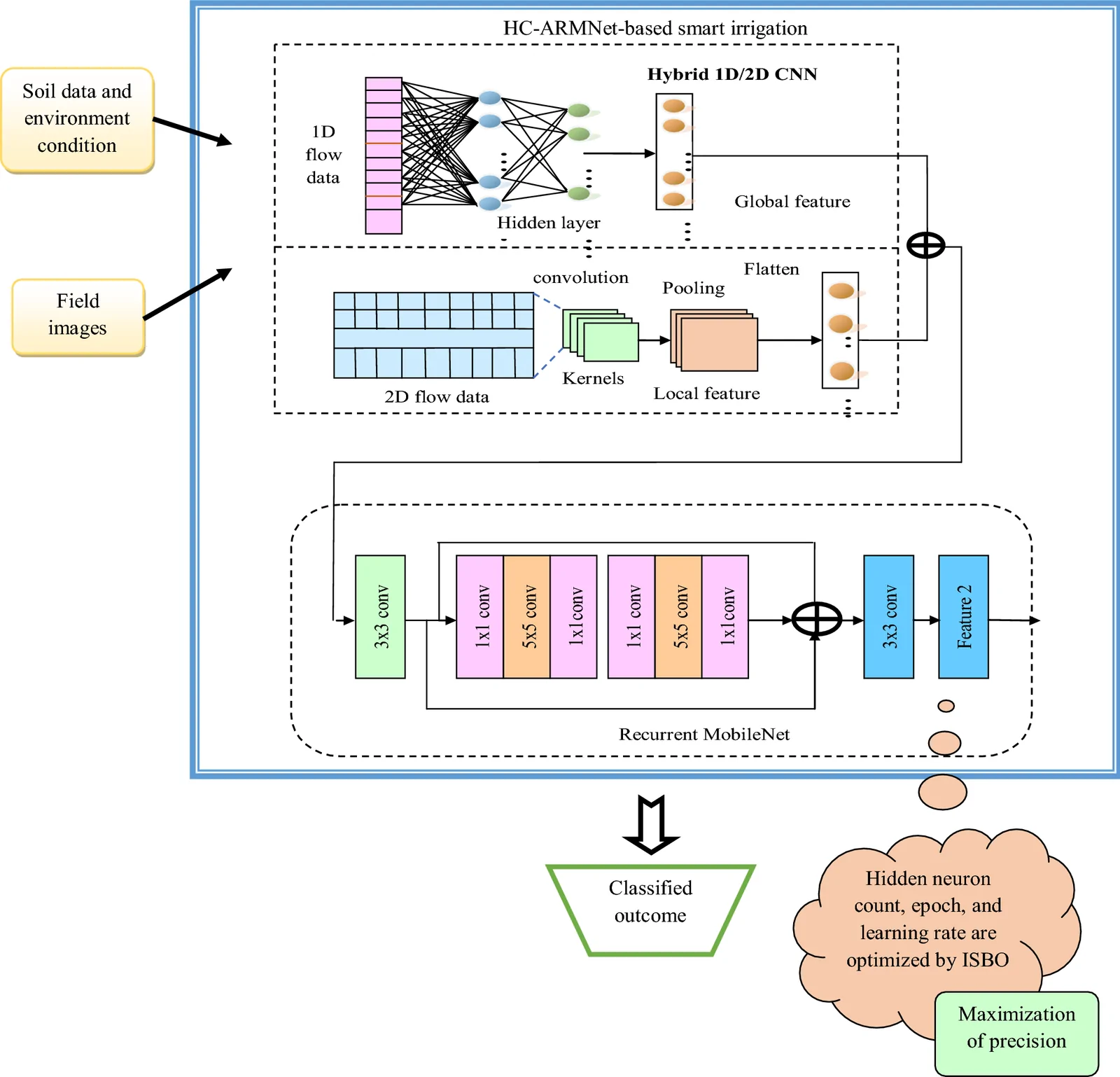
Structural diagram of the HC-ARMNet-aided smart irrigation process for an IoT-based smart farming framework.

Nowadays, precision agriculture has achieved high attention because the emerging world population requires water and food. The farmers need water and suitable land to satisfy this requirement. Because of the constrained existence of both resources, the farmers demand a solution that transforms the way they work. Accurate irrigation is the answer to provide better, bigger, and highly profitable yields with lower resources. Some machine learning-aided irrigation techniques have been developed to utilize water very effectively. Because of the limited learning capacity of these techniques, the machine learning strategies can’t be applied in unexpected climates. Deep learning-based techniques were also invented in the smart irrigation field. However, these techniques fail to provide satisfactory solutions. Therefore, this work suggested an effective HC-ARMNet mechanism for performing the smart irrigation process. In this process, from the blockchain data $A_{i}^{Bl}$, the secured field images are subjected as input to the 2D convolution, whereas the soil and environment data are forwarded as input to the 1D convolution. From this hybrid convolution process, the rich features are extracted and given to the recurrent MobileNet approach that effectively performs the smart irrigation process. Here also, the same ISBO is utilized for optimizing the parameters of the recurrent MobileNet for providing precise outcomes. Finally, the classified outcome is obtained by the developed HC-ARMNet technique. The structural diagram of the developed HC-ARMNet-based smart irrigation system is displayed in Fig. 8.

## Results and discussions

### Experimental Setup

The IoT-based smart farming system, by utilizing the blockchain mechanism, was developed in Python, and satisfactory solutions were achieved. The designed ISBO leveraged 10 populations and 50 iterations. Moreover, the considered chromosome length of the ISBO was 3. The existing algorithms, such as Cuttlefish Optimization (CO)^30^, Gannet Optimization Algorithm (GaOA)^31^, Golf Optimization Algorithm (GOA)^32^, and SBO^26^, were employed in the performance analysis. Additionally, classical techniques such as Deep Neural Network (DNN)^33^, 1D Convolutional Neural Network (1DCNN)^34^, Long Short Term Memory (LSTM)^35^, and HC-RMNet^28^^29,36^ were considered for the performance verification of the implemented model. Table 2 gives the Ethereum blockchain’s configuration parameters.

**Table 2:** Ethereum blockchain’s configuration parameters.

Parameter	Value/Description
Blockchain type	Private consortium ethereum network
Consensus mechanism	Proof-of-authority (PoA) – clique protocol
Number of validator nodes	10
Average block size	1 MB
Average block generation time	5 seconds
Transaction throughput	~150–200 transactions per second (TPS)
Smart contract language	Solidity
Virtual machine	Ethereum virtual machine (EVM)
Hashing algorithm	Keccak-256
Digital signature algorithm	Elliptic curve digital signature algorithm (ECDSA)
Data storage mechanism	On-chain metadata; Off-chain data via IPFS
Encryption scheme	AES-256 for IoT–Blockchain communication
Gas limit per block	12,500,000 units
Network ID	1515 (custom private network)
Average transaction confirmation time	8–10 seconds
Smart contract functions	Data validation, pest alert automation, and irrigation control
Client software	Geth (Go Ethereum v1.13)
Operating environment	Ubuntu 22.04 LTS with dockerized nodes
Node hardware setup	8 GB RAM, 4 vCPUs, 100 GB SSD per node
Connectivity	MQTT–HTTP bridge for IoT to blockchain interface

#### Code availability

The implementation code, trained models, and experimental configurations used are publicly available in: “https://github.com/sumanthvmani/-Pest-Detection-and-Smart-Irrigation”. The repository contains all necessary instructions and dependencies required to reproduce the reported experimental results. The code has been made available in accordance with the journal’s reproducibility and editorial policies.

### Performance metrics

The evaluation metrics used in the designed IoT-aided smart farming system are explained as follows.

*Precision:* It is shown in Eq. (30).

*Accuracy:*

31 $$A = \frac{dx + gx}{{dx + gx + jx + lx}}$$

*FNR:*

32 $$FNR = \frac{lx}{{dx + lx}}$$

*FPR:*

33 $$FPR = \frac{jx}{{jx + dx}}$$

*Sensitivity:*

34 $$Sen = \frac{dx}{{dx + gx}}$$

*Specificity:*

35 $$spec = \frac{jx}{{jx + lx}}$$

*Balanced accuracy (BA):*

36 $$BA = \frac{lx + gx}{2}$$

*Book maker (BM):*

37 *BM = dx + gx - 1*

*F1 score:*

38 $$F1 - Score = 2 \times \frac{gx \times jx}{{gx + jx}}$$

*NPV:*

39 $$NPV = \frac{dx}{{dx + lx}}$$

*MAE:*

40 $$MAE = \frac{{\sum\limits_{q = 1}^{Q} {\left( {d_{q} - f_{q} } \right)} }}{E}$$

*MASE:*

41 $$MASE = \frac{1}{E}\sum\limits_{q = 1}^{Q} {\left( {\frac{{f_{q} - d_{q} }}{{f_{q} }}} \right)}$$

*MSE:*

42 $$MSE = \frac{1}{E}\sum\limits_{q = 1}^{Q} {\left( {d_{q} - f_{q} } \right)}$$

*One-norm:*

43 $$ON = \sum\limits_{q} {\left( {G_{q} } \right)}$$

*RMSE:*

44 $$RMSE = \sqrt {\frac{{\sum\limits_{q = 1}^{Q} {\left( {d_{q} - f_{q} } \right)^{2} } }}{E}}$$

*SMAPE:*

45 $$SMAPE = \frac{100\% }{E}\sum\limits_{q = 1}^{Q} {\left( {\frac{{d_{q} - f_{q} }}{{{\raise0.7ex\hbox{${d_{q} + f_{q} }$} \!\mathord{\left/ {\vphantom {{d_{q} + f_{q} } 2}}\right.\kern-0pt} \!\lower0.7ex\hbox{$2$}}}}} \right)}$$

*Two-norm:*

46 $$TN = \left( {\sum\limits_{q = 1}^{Q} {G_{q}^{2} } } \right)^{{{\raise0.7ex\hbox{$1$} \!\mathord{\left/ {\vphantom {1 2}}\right.\kern-0pt} \!\lower0.7ex\hbox{$2$}}}}$$

*Infinity-norm:*

47 $$IN = \mathop {\max }\limits_{1 \le q \le Q} \left( {G_{q} } \right)$$

*FDR:*

48 $$FDR = \frac{jx}{{dx + jx}}$$

Here, the attributes $gx\,\,\,\,and\,\,\,lx$ are true negative and true positive values. The factor *G* specifies the matrix, and the overall size is *E*. The observed and the predicted values are given as $f\,\,\,\,and\,\,\,\,d$ accordingly. Then, the data point is given as *q*.

### Convergence investigation of the developed ISBO

**Fig. 9:**
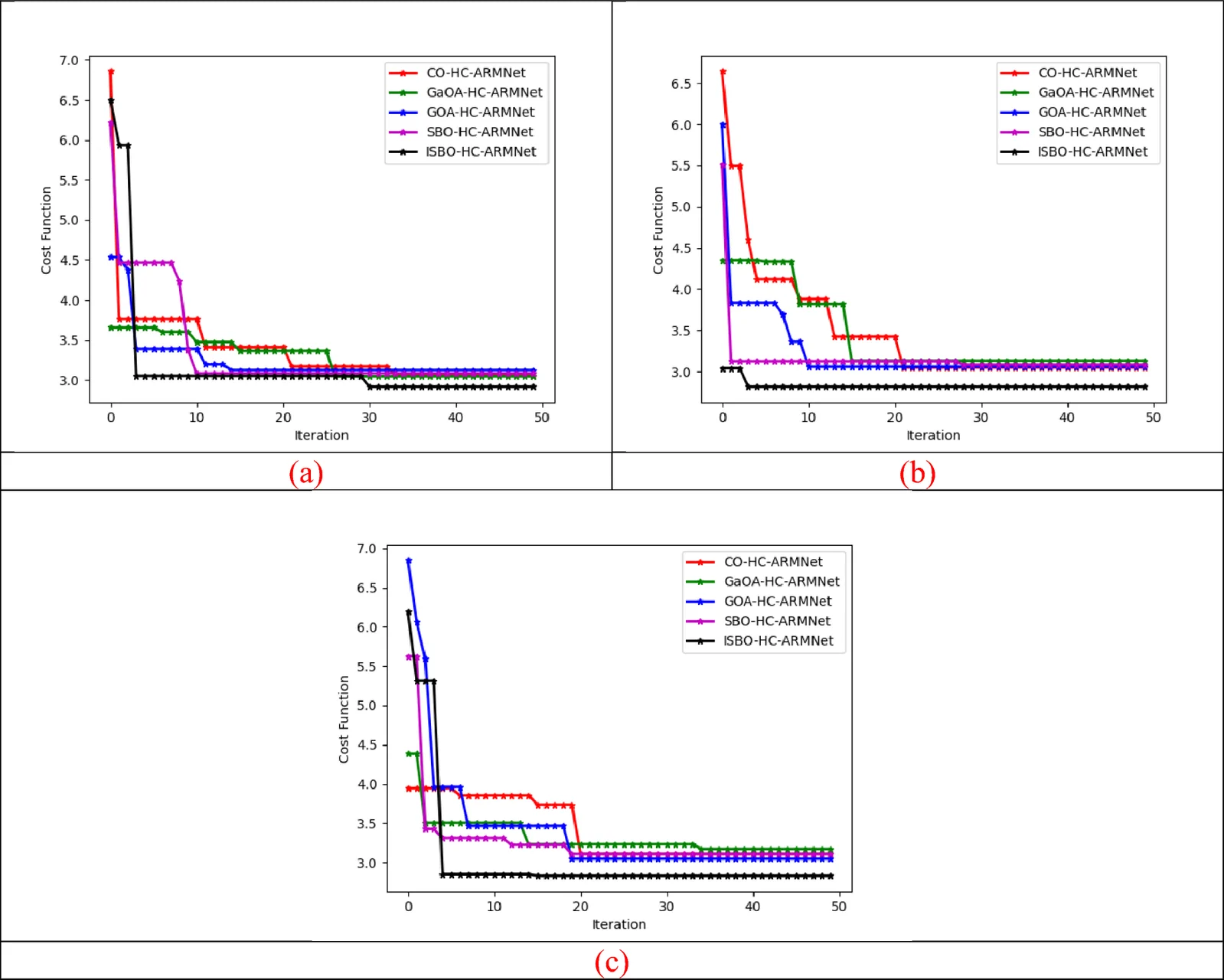
Convergence assessment of implemented ISBO over other recent algorithms in terms of “(**a**) Plant disease prediction dataset, (**b**) Pest detection dataset, and (**c**) Smart irrigation dataset”.

The designed ISBO algorithm’s convergence is assessed over distinct existing algorithms using iteration values. This experiment is given in Fig. 9 for three data sources. When the iteration is 15 in Fig. 9 (a), the designed ISBO’s cost function is decreased by 12.6% ofCO-HC-ARMNet, 16% of GaOA-HC-ARMNet, 10.6% of GOA-HC-ARMNet, and 11.3% of SBO-HC-ARMNet, respectively. Also, for other datasets, the implemented ISBO gives very low convergence values. Hence, it has been elaborated that the recommended ISBO attained very low-cost function values. Therefore, it has been reported that the implemented ISBO obtained more convergence values than the other recent algorithms.

### Statistical investigation of developed ISBO

By considering the three effective datasets, the developed ISBO model’s statistical validation is conducted. Table 3 displays this analysis for the developed ISBO over existing algorithms. Effective statistical measures are involved in this validation. When using the median measure in the pest detection dataset, the developed ISBO shows 8.18%, 11.38%, 8.54%, and 11.03% improved outcomes than the existing algorithms, such as CO-HC-ARMNet, GaOA-HC-ARMNet, GOA-HC-ARMNet, and SBO-HC-ARMNet, respectively. Therefore, it is portrayed that the recommended ISBO provides higher quality outcomes than the existing algorithms.

**Table 3:** Statistical investigation of the ISBO algorithm over classical algorithms.

Terms	CO- HC-ARMNet^30^	GaOA- HC-ARMNet^31^	GOA- HC-ARMNet^32^	SBO- HC-ARMNet^26^	ISBO- HC-ARMNet
Plant disease prediction dataset					
Best	3.055864524	3.045731547	3.123609171	3.084257282	2.916292156
Worst	6.859584658	3.654494265	4.536083847	6.21666589	6.50301696
Mean	3.372087068	3.276603598	3.252225975	3.369530801	3.181906278
Median	3.169143382	3.364928796	3.123609171	3.084257282	3.051739043
Standard deviation	0.563648633	0.238578454	0.326352858	0.641257204	0.749060256
Pest detection dataset					
Best	3.047464698	3.130297859	3.058053871	3.08247244	2.814759644
Worst	6.645286024	4.351412591	6.000695514	5.514550333	3.04097029
Mean	3.482205649	3.431435158	3.23509672	3.154107014	2.828332283
Median	3.047464698	3.130297859	3.058053871	3.1250521	2.814759644
Standard deviation	0.74189853	0.481063717	0.475543279	0.337857337	0.053722052
Smart irrigation dataset					
Best	3.107812549	3.170780501	3.046744351	3.107841779	2.828474862
Worst	3.943751815	4.385315521	6.849361502	5.625895428	6.194964972
Mean	3.405070586	3.323672308	3.407658849	3.26983146	3.049576501
Median	3.107812549	3.2311267	3.046744351	3.107841779	2.828474862
Standard deviation	0.367290287	0.252717196	0.761602424	0.489768802	0.740351409

### Performance investigation of the plant disease classification process

**Fig. 10:**
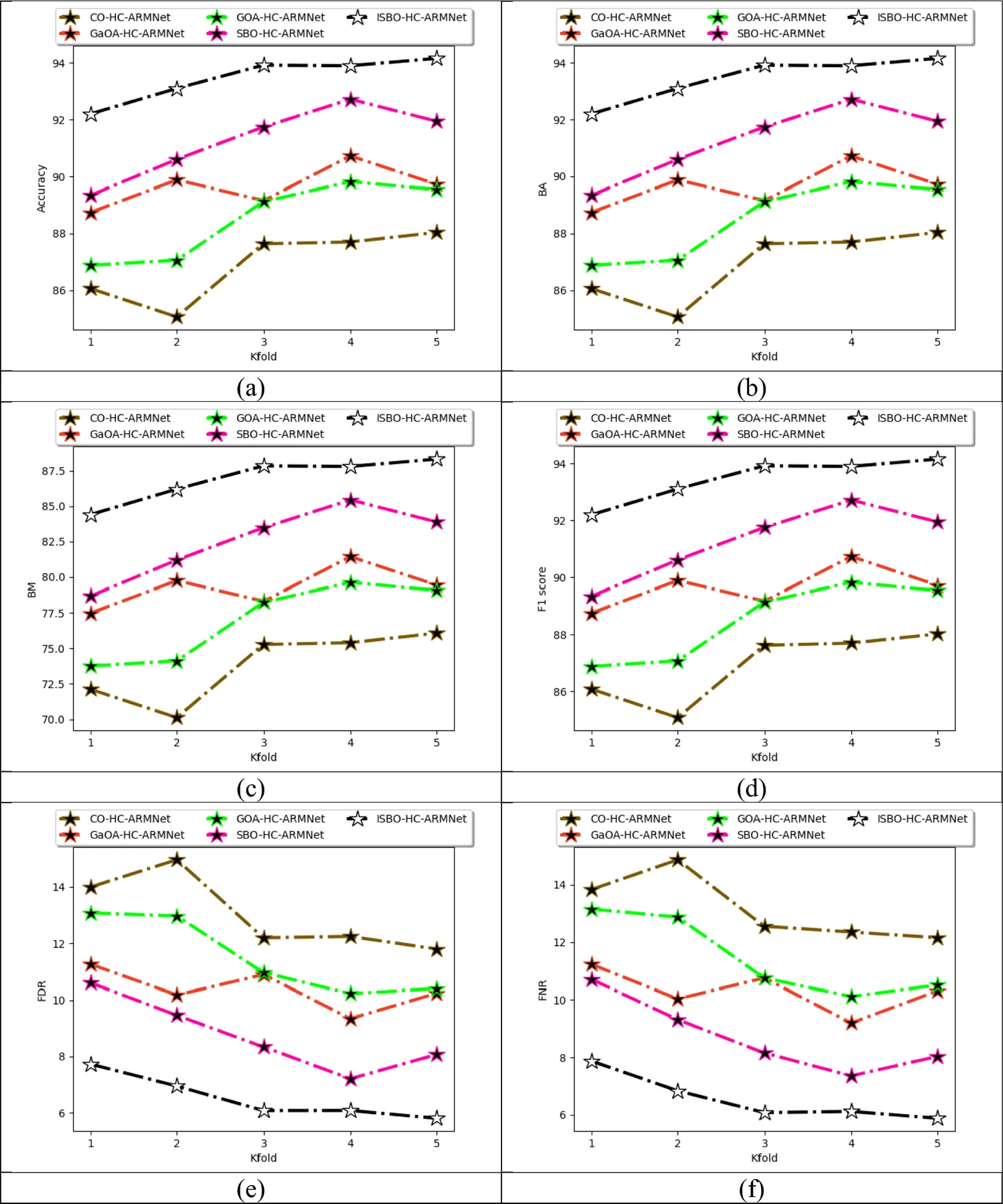

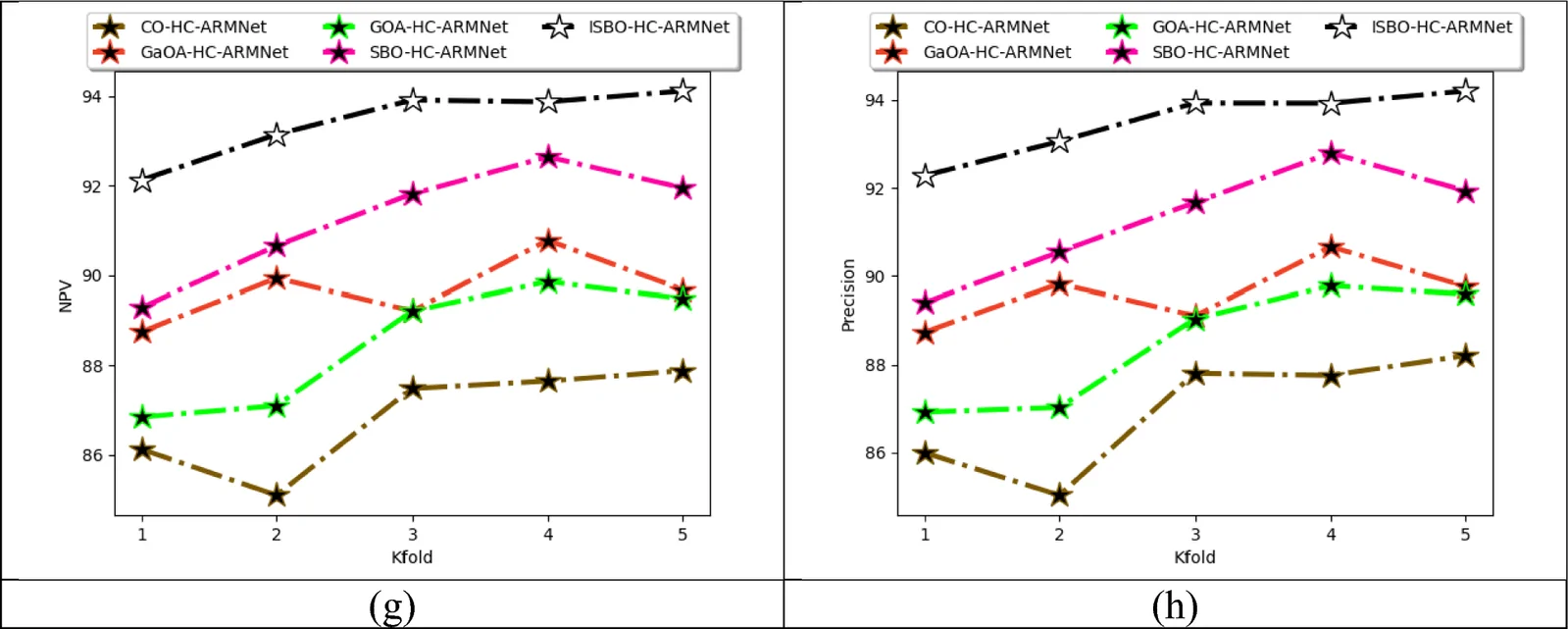
Performance investigation of implemented plant disease classification process over traditional algorithms in terms of “(**a**) Accuracy, (b) BA, (**c**) BM, (**d**) F1 score, (**e**) FDR, (**f**) FNR, (**g**) NPV, and (**h**) Precision”.

**Fig. 11:**
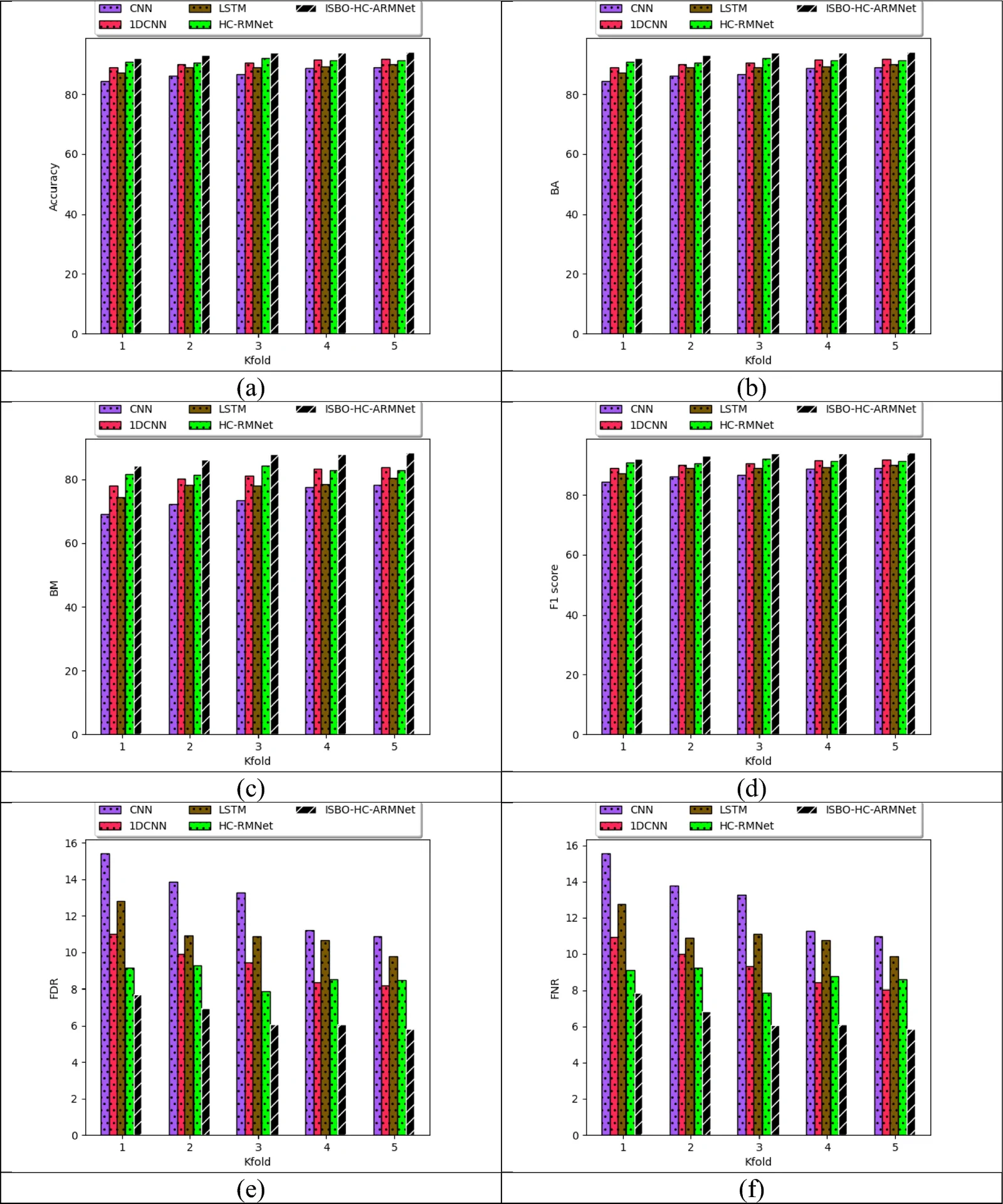

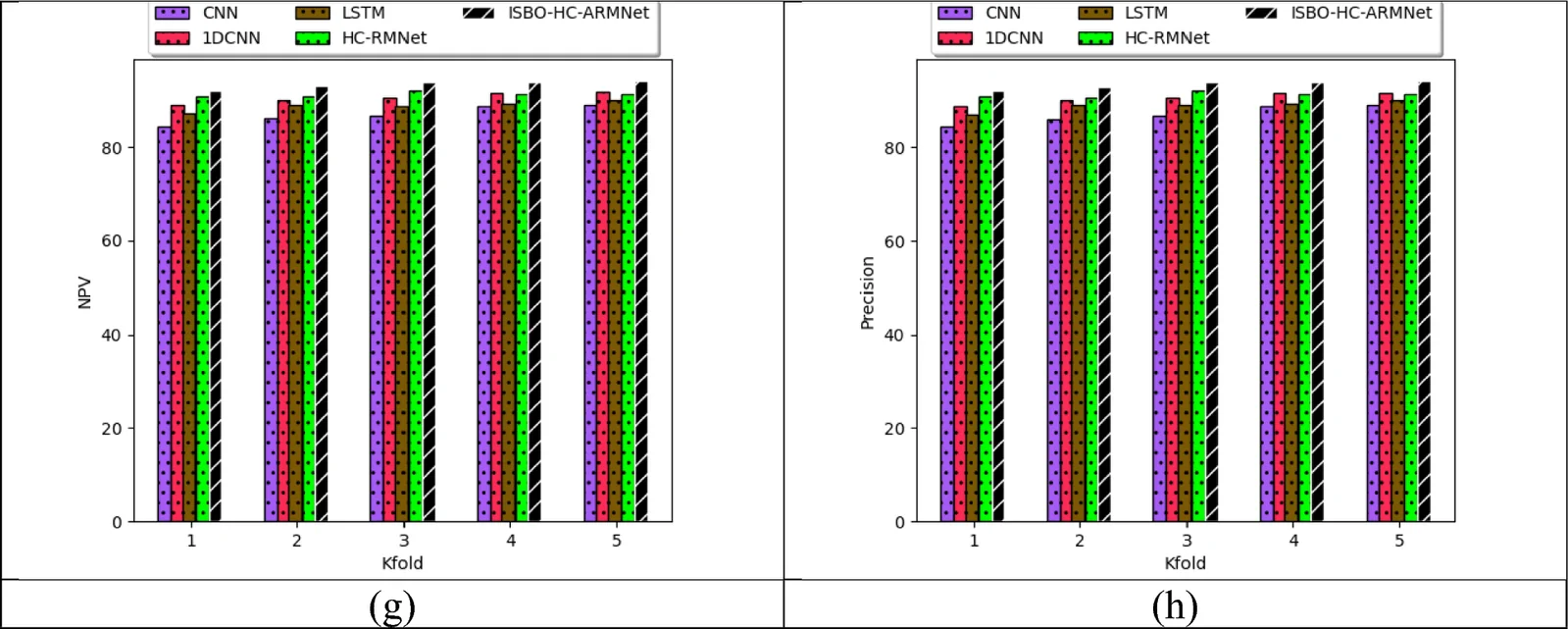
Performance investigation of implemented plant disease classification process over traditional classifiers in terms of “(**a**) Accuracy, (**b**) BA, (**c**) BM, (**d**) F1 score, (**e**) FDR, (**f**) FNR, (**g**) NPV, and (**h**) Precision”.

By utilizing the plant disease prediction dataset, the performance of the plant disease classification task is analyzed. The performance investigation of the plant disease classification process is carried out in Fig. 10 and Fig. 11 over traditional algorithms and classifiers. The k-fold factor is used for estimating the plant disease classification process. For the 4^th^ k-fold value in Fig. 10 (a), the designed plant disease classification process obtained 6.5%, 4.05%, 4.58%, and 1.91% higher accuracy than the classical algorithms such as CO-HC-ARMNet, GaOA-HC-ARMNet, GOA-HC-ARMNet, and SBO-HC-ARMNet, accordingly. Also, when focusing on the classifier-based analysis, the developed plant disease classification process provided improved solutions. Therefore, it has been proven that the implemented plant disease classification task attained highly accurate solutions than the traditional algorithms and classifiers.

### Performance investigation of the pest detection process

**Fig. 12:**
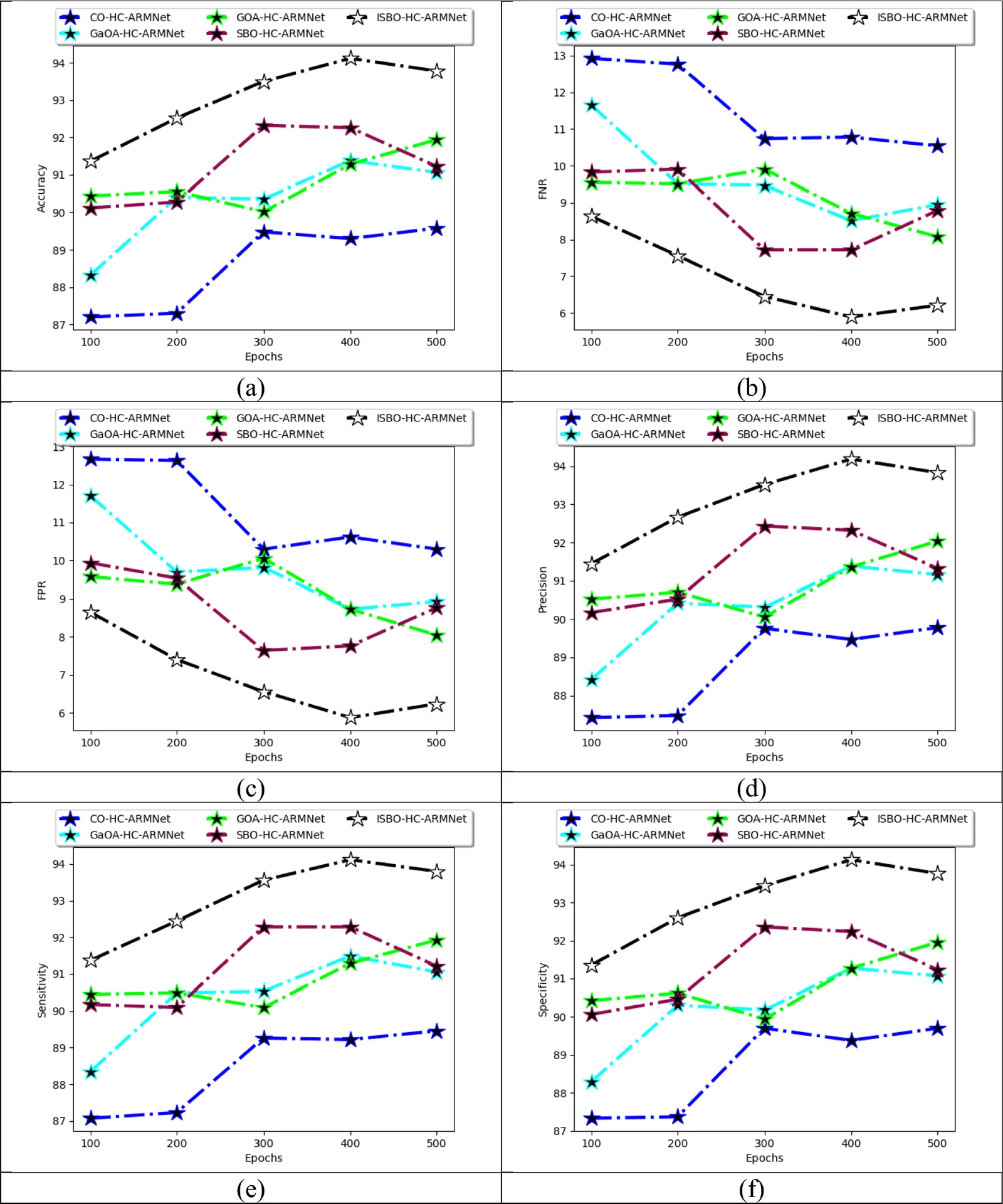
Performance validation of the pest detection process over traditional algorithms in terms of “(**a**) Accuracy, (**b**) FNR, (**c**) FPR, (**d**) Precision, (**e**) Sensitivity, and (**f**) Specificity”.

**Fig. 13:**
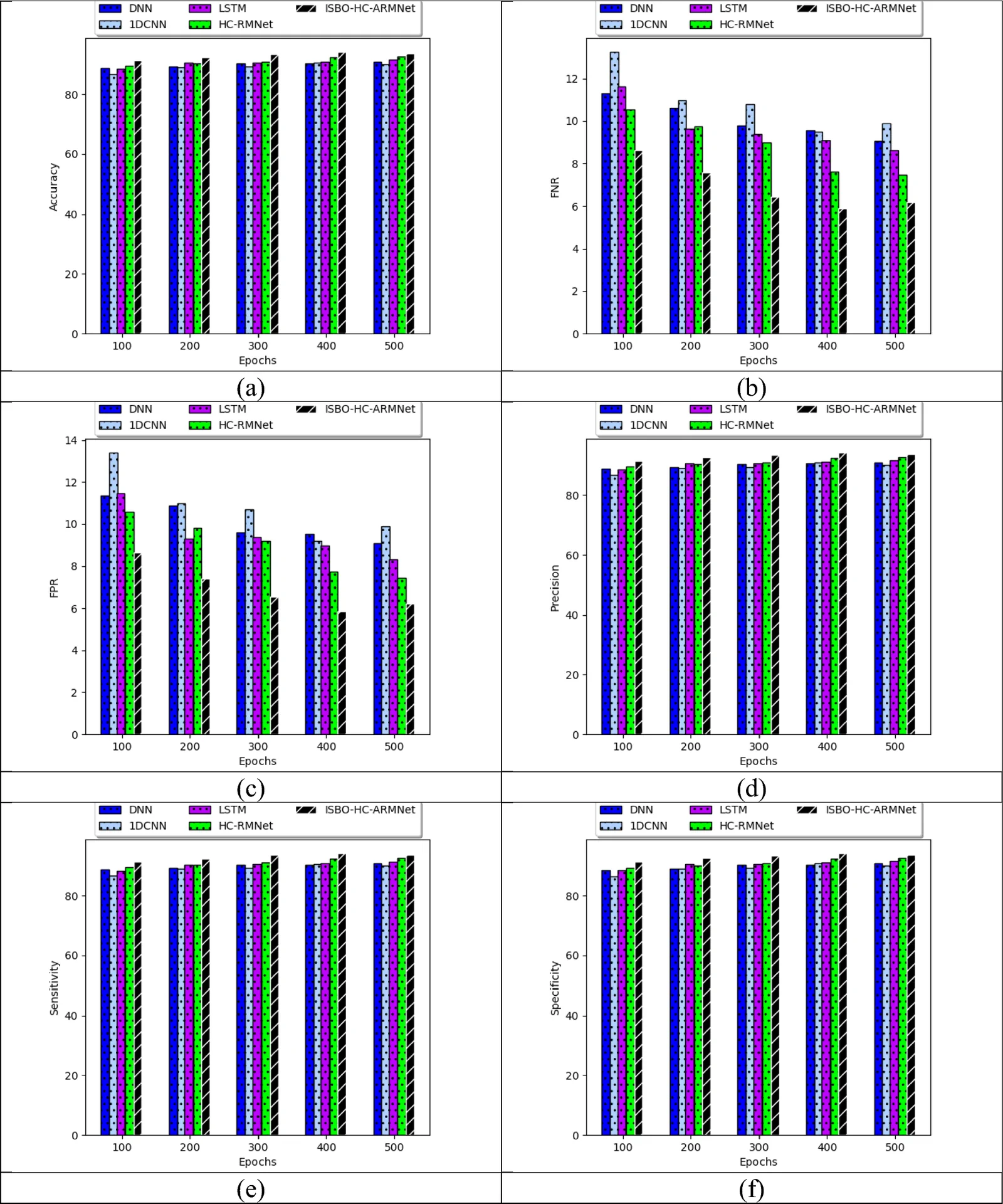
Performance investigation of implemented pest detection process over traditional classifiers in terms of “(**a**) Accuracy, (**b**) FNR, (**c**) FPR, (**d**) Precision, (**e**) Sensitivity, and (**f**) Specificity”.

The pest detection method’s performance is validated in Fig. 12 and Fig. 13 over existing algorithms and classifiers. By utilizing the pest detection dataset and effective performance measures, this validation is performed. Moreover, by considering the epoch values, this analysis performed. From Fig. 13 (d), the designed pest detection model’s precision is enhanced by 10.52% of DNN, 9.47% of 1DCNN, 8.42% of LSTM, and 5.26% of HC-RMNet, respectively, for the 400^th^ epoch value. Also, for algorithm-based evaluation, the implemented pest detection process gives higher performance rates. For algorithm-based evaluation, the designed pest detection process also shows higher performance rates. Hence, it is showed that the introduced pest detection process achieved more effective solutions than the traditional models.

### Performance investigation of a smart irrigation system

**Fig. 14:**
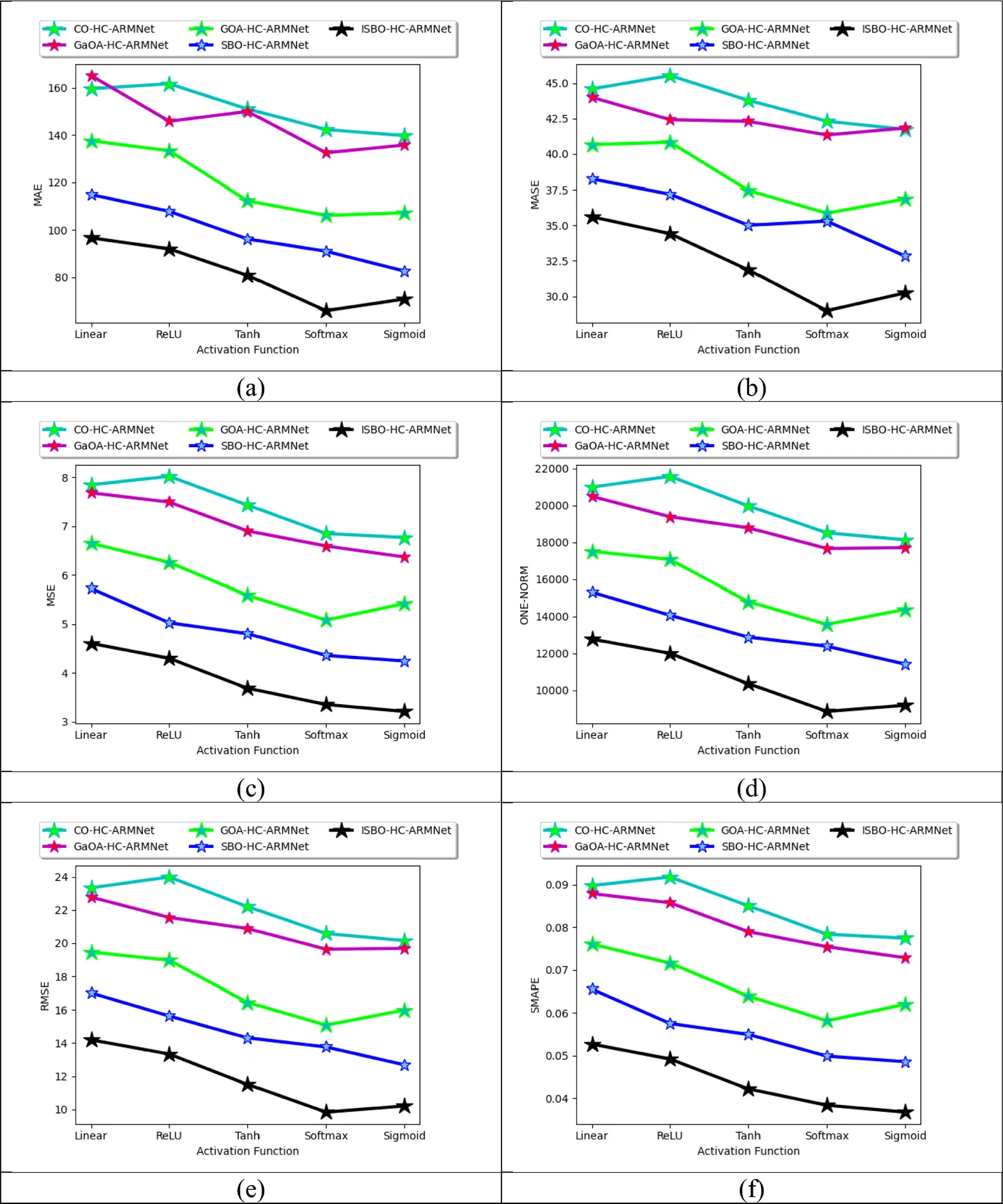

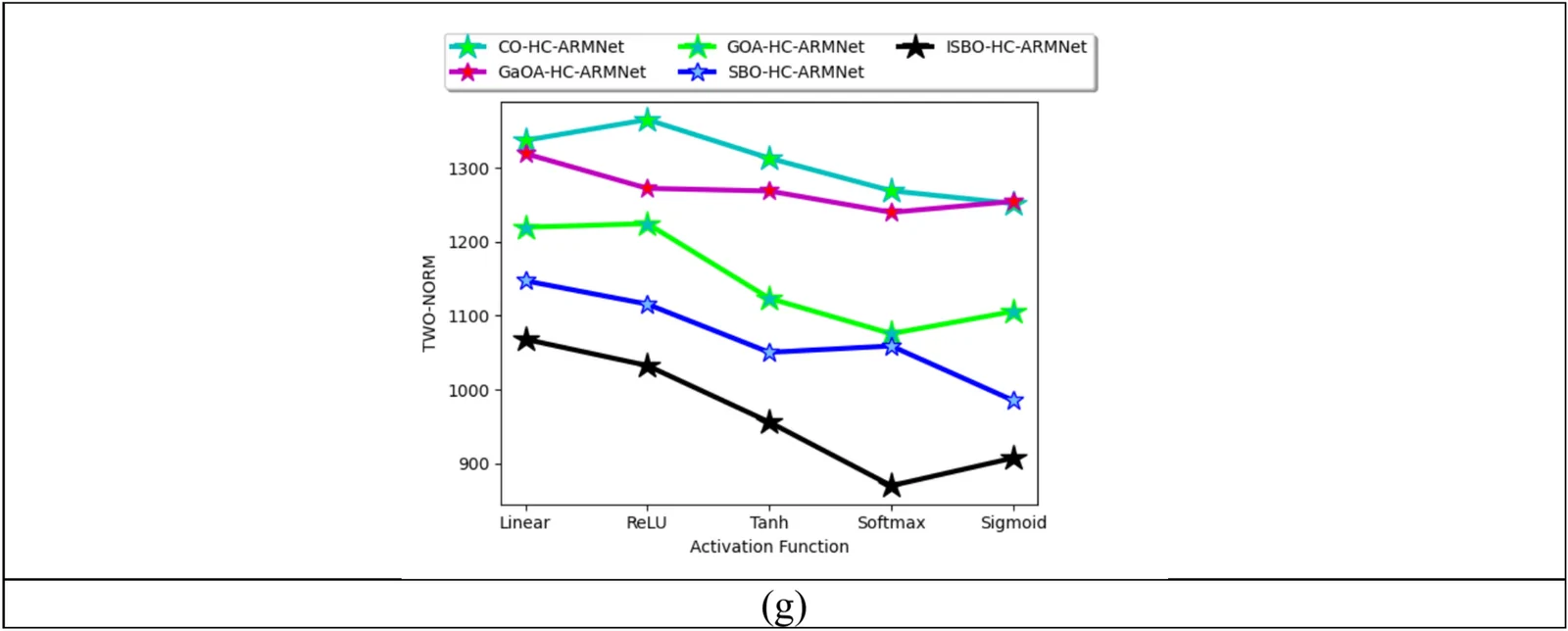
Performance investigation of implemented smart irrigation system over traditional algorithms in terms of “(**a**) MAE, (**b**) MASE, (c) MSE, (**d**) One-norm, (**e**) RMSE, (**f**) SMAPE, and (**g**) Two-norm”.

**Fig. 15:**
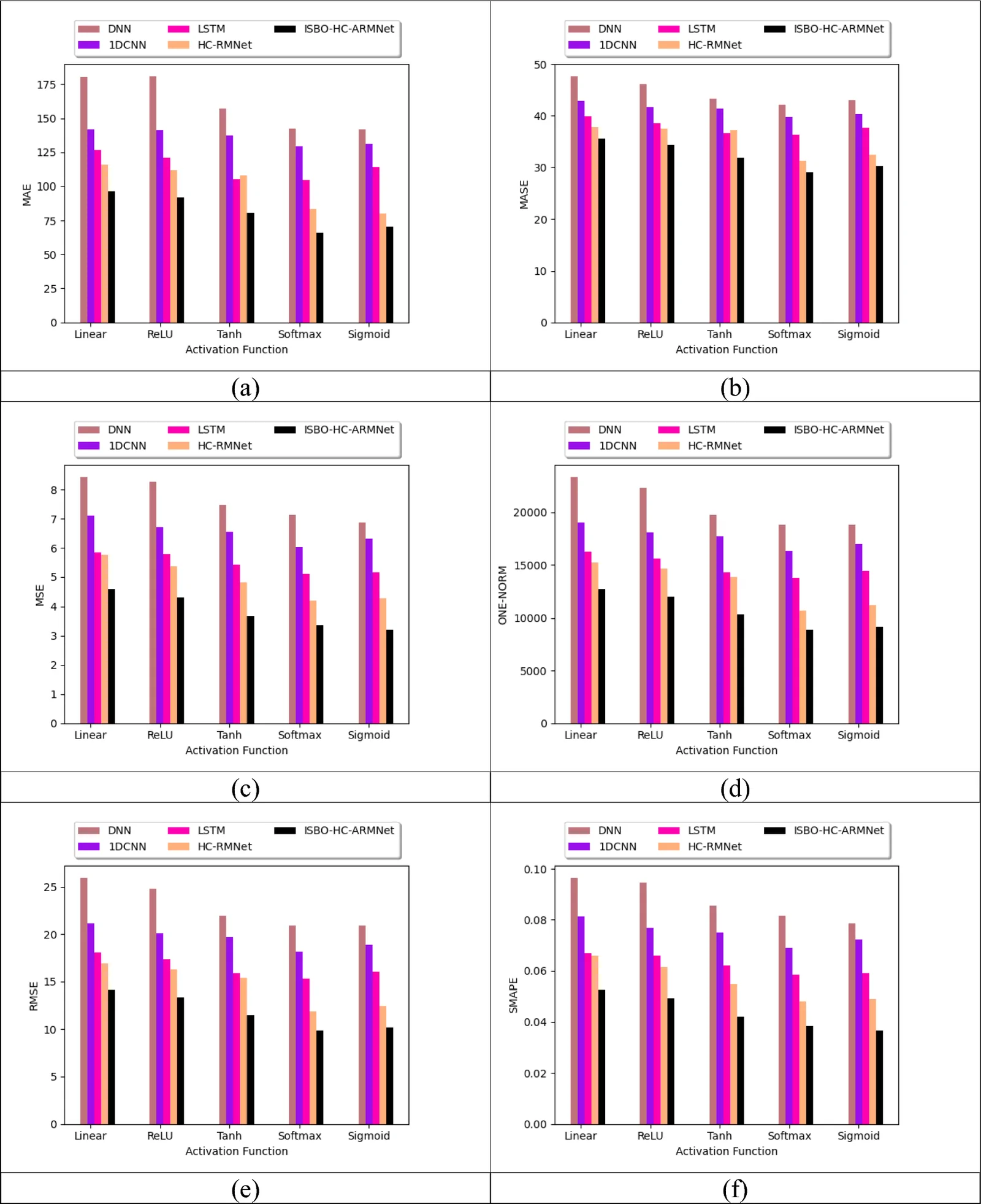

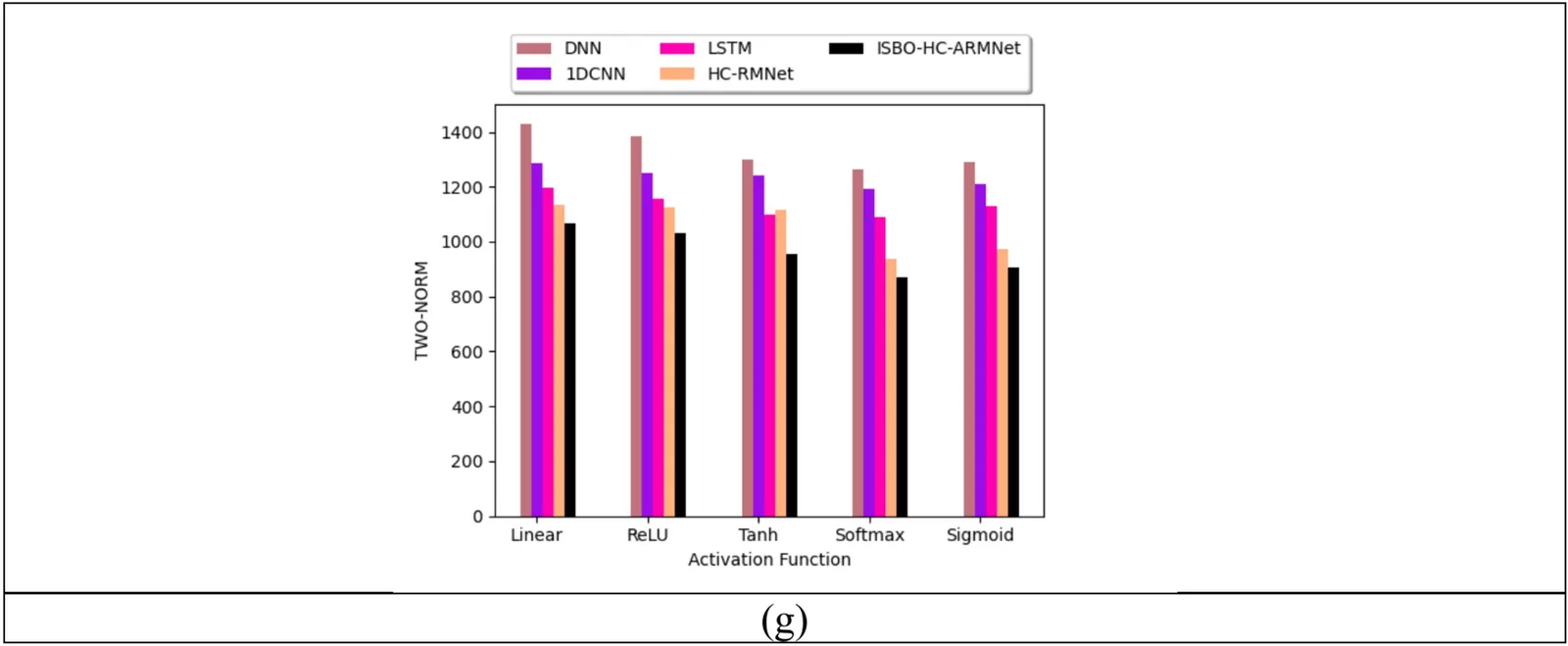
Performance investigation of implemented smart irrigation system over traditional classifiers in terms of “(**a**) MAE, (**b**) MASE, (**c**) MSE, (**d**) One-norm, (**e**) RMSE,(**f**) SMAPE, and (**g**) Two-norm”.

The smart irrigation process’s efficiency is examined over traditional algorithms and classifiers. This experiment is visualized in Fig. 14 and Fig. 15 using numerous error measures. By employing the smart irrigation dataset and activation functions, this experiment is conducted. When taking the sigmoid activation function in Fig. 14 (b), the developed smart irrigation process achieved 41.6%, 41.6%, 25%, and 10% lower MASE than the conventional algorithms, such as CO-HC-ARMNet, GaOA-HC-ARMNet, GOA-HC-ARMNet, and SBO-HC-ARMNet, correspondingly. For classifier-based evaluation, the implemented smart irrigation process also gives very low error values. Hence, it has been proven that the introduced smart irrigation process attained very low error values than the existing algorithms and classifiers.

### Overall comparative investigation of the plant disease classification process

The comparative examination of the plant disease classification task is shown in Table 4 over existing methods and classifiers. By employing the 5^th^ K-fold value, this evaluation is conducted. The F1-score of the designed plant disease classification process is improved by 5.39% of DNN, 2.41% of 1DCNN, 4.22% of LSTM, and 2.86% of HC-RMNet appropriately. The algorithm-based evaluation also displayed the enhanced efficiency rates of the developed process. Thus, it has been ensured that the suggested plant disease classification operation achieved superior outcomes than the previous algorithms and classifiers.

**Table 4:** Overall comparative investigation of the introduced plant disease classification process over classical algorithms and classifiers using the 5^th^ K-fold value.

Algorithm-based evaluation					
Terms	CO- HC-ARMNet^30^	GaOA- HC-ARMNet^31^	GOA- HC-ARMNet^32^	SBO- HC-ARMNet^26^	ISBO- HC-ARMNet
Accuracy	88.04	89.72	89.54	91.94	94.16
Precision	88.20224719	89.76	89.59583834	91.92968438	94.2
FNR	12.15027978	10.3117506	10.51159073	8.033573141	5.87529976
NPV	87.87878788	89.68	89.48420632	91.95034041	94.12
FDR	11.79775281	10.24	10.40416166	8.070315621	5.8
F1 score	88.02563076	89.72411036	89.54209158	91.94805195	94.16233507
BA	88.04015235	89.72002542	89.54004131	91.93997884	94.16002826
BM	76.08030469	79.44005084	79.08008261	83.87995768	88.32005652

**Classifier-based evaluation**					
**Terms**	**DNN**^33^	**1DCNN**^34^	**LSTM**^35^	HC-RMNet^28,29,36^	**ISBO-** HC**-**ARMNet
Accuracy	89.08	91.88	90.18	91.46	94.16
Precision	89.12	91.81963288	90.23609444	91.51660664	94.2
FNR	10.95123901	8.033573141	9.872102318	8.5931255	5.87529976
NPV	89.04	91.94065758	90.12395042	91.40343862	94.12
FDR	10.88	8.180367119	9.763905562	8.483393357	5.8
F1 score	89.08436625	91.89297125	90.18196361	91.46170766	94.16233507
BA	89.08002501	91.8799308	90.18004172	91.46004253	94.16002826
BM	78.16005002	83.75986161	80.36008343	82.92008507	88.32005652

### Overall comparative investigation of the pest detection process

By using the 500^th^ epoch count, the implemented pest detection operation’s effectiveness is analyzed and displayed in Table 5 over existing algorithms and models. The sensitivity of the designed pest detection process is 4.62%, 2.92%, 1.99%, and 2.76% enhanced by CO-HC-ARMNet, GaOA-HC-ARMNet, GOA-HC-ARMNet, and SBO-HC-ARMNet, accordingly. For classifier-based evaluation, the designed model provides improved outcomes. Thus, it has been confirmed that the implemented pest detection process shows outstanding solutions than the classical algorithms and classifiers.

**Table 5:** Overall comparative investigation of the introduced pest detection process over prior algorithms and classifiers using the 500^th^ epoch value.

Algorithm-based evaluation					
Terms	CO-HC-ARMNet^30^	GaOA-HC-ARMNet^31^	GOA-HC-ARMNet^32^	SBO-HC-ARMNet^26^	ISBO- HC-ARMNet
Accuracy	89.58	91.06	91.94	91.22	93.78
Sensitivity	89.45902944	91.05011933	91.92521877	91.20922832	93.7947494
Specificity	89.70233307	91.06999195	91.95494771	91.230893	93.76508447
Precision	89.78043912	91.15890084	92.0350458	91.31819992	93.83207322
FPR	10.29766693	8.930008045	8.045052293	8.769106999	6.234915527
FNR	10.54097056	8.949880668	8.074781225	8.790771679	6.205250597

Classifier-based evaluation					
Terms	DNN^33^	1DCNN^34^	LSTM^35^	HC-RMNet^28^^29^^36^	ISBO-HC-ARMNet
Accuracy	90.92	90.1	91.52	92.54	93.78
Sensitivity	90.93078759	90.09546539	91.36833731	92.52187749	93.7947494
Specificity	90.90909091	90.10458568	91.67337088	92.55832663	93.76508447
Precision	91.00318471	90.20310633	91.73322684	92.63241736	93.83207322
FPR	9.090909091	9.89541432	8.326629123	7.441673371	6.234915527
FNR	9.069212411	9.904534606	8.631662689	7.478122514	6.205250597

### Overall comparative investigation of the smart irrigation system

The developed smart irrigation process’s comparative evaluation is given in Table 6 over traditional algorithms and classifiers. By utilizing the sigmoid activation function, the performance is examined. The MSE of the developed smart irrigation process achieved 11.4%, 9.6%, 6%, and 3.3% lower values than the existing classifiers, such as DNN, 1DCNN, LSTM, and HC-RMNet, respectively. For algorithm-based analysis, the developed smart irrigation process also provides lower error rates. Thus, it has been confirmed that the developed smart irrigation process provides more satisfactory solutions than the conventional algorithms and classifiers.

**Table 6:** Overall comparative investigation of the introduced smart irrigation process over prior algorithms and classifiers using the sigmoid activation function.

Algorithm-based evaluation					
Terms	CO-HC-ARMNet^30^	GaOA-HC-ARMNet^31^	GOA- HC-ARMNet^32^	SBO-HC-ARMNet^26^	ISBO-HC-ARMNet
MSE	6.768592287	6.369218156	5.421435334	4.247584541	3.212434289
SMAPE	0.077432206	0.072850378	0.062013082	0.048585784	0.03674325
MASE	41.73485088	41.82928267	36.86029179	32.8363112	30.23814736
MAE	139.775431	135.7582744	107.2554801	82.61386115	70.71842622
RMSE	20.15333333	19.68	15.98333333	12.68777778	10.19888889
ONE-NORM	18138	17712	14385	11419	9179
TWO-NORM	1252.045526	1254.87848	1105.808754	985.0893361	907.1444207
INFINITY-NORM	125	125	124	125	125

Classifier-based evaluation					
Terms	DNN^33^	1DCNN^34^	LSTM^35^	HC-RMNet^28^^,^^29,36^	ISBO- HC-RMNet
MSE	6.874445088	6.318466041	5.166308521	4.275990728	3.212434289
SMAPE	0.078633129	0.072278359	0.059088844	0.04891148	0.03674325
MASE	42.97501858	40.29026626	37.67790393	32.46829222	30.23814736
MAE	142.111029	131.0120663	114.0021386	80.3418332	70.71842622
RMSE	20.92111111	18.86777778	16.07555556	12.47666667	10.19888889
ONE-NORM	18829	16981	14468	11229	9179
TWO-NORM	1289.250557	1208.707988	1130.337118	974.0487667	907.1444207
INFINITY-NORM	125	125	125	125	125

### Comparative analysis

The comparative analysis of the designed ISBO-HC-ARMNet is presented in Table 7, which is designed to classify the plant disease, detect the pests and smart irrigation processes. Here, the designed ISBO-HC-ARMNet is contrasted with the existing methods using various performance metrics. When considering the classification phase of plant disease, the designed ISBO-HC-ARMNet achieves 94.16% accuracy, while in the pest detection phase, the designed ISBO-HC-ARMNet achieves 93.78% accuracy. In addition, the designed ISBO-HC-ARMNet’s MSE rate is 3.21%. All these measures are superior to the traditional methods, such as CHSS, Trust-based decentralized blockchain, RT and RNECB in all the phases. Hence, it is proven that the recommended ISBO-HC-ARMNet is promising in the presented IoT-driven smart farming system than the traditional approaches.

**Table 7:** Comparative analysis of the designed IoT-driven smart farming system.

Terms	CHSS^18^	Trust-based decentralized blockchain^21^	RT^24^	RNECB^25^	ISBO- HC-ARMNet
Plant disease classificatio					
Accuracy	87.26	88.93	89.15	91.42	94.16
Precision	87.38	89.02	89.18	91.47	94.20
FNR	12.74	11.07	10.85	8.58	5.87
NPV	87.14	88.89	89.07	91.45	94.12
FDR	12.62	10.98	10.82	8.53	5.80
F1 score	87.24	88.96	89.14	91.44	94.16
BA	87.28	88.94	89.13	91.46	94.16
BM	74.56	77.88	78.26	82.92	88.32
Pest detection					
Accuracy	88.72	90.35	90.82	91.26	93.78
Sensitivity	88.64	90.29	90.76	91.2	93.79
Specificity	88.83	90.41	90.89	91.33	93.77
Precision	88.91	90.48	90.95	91.39	93.83
FPR	11.17	9.59	9.11	8.67	6.23
FNR	11.36	9.71	9.24	8.8	6.21
Smart irrigation					
MSE	6.924513	6.512847	5.694325	4.516237	3.212434
SMAPE	0.0798	0.0743	0.0645	0.0502	0.03674
MASE	42.1842	41.9728	37.1385	33.2156	30.2381
MAE	141.562	136.847	109.483	84.279	70.7184
RMSE	20.478	19.934	16.241	12.954	10.1989
ONE-NORM	18296	17863	14542	11582	9179
TWO-NORM	1261.73	1259.86	1118.54	992.67	907.144
INFINITY-NORM	125	124	124	125	125

### Performance improvement of ISBO-HC-ARMNet before and after class imbalance

The results in Table 8 prove that the SMOTE integrated with a class-weighted loss function highly improved the performance of the approach across all predictive tasks, such as plant disease classification, pest detection and smart irrigation prediction in the IoT-blockchain-based smart farming model. Before balancing, the datasets suffered from skewed class distributions, resulting in lower F1 score, recall and higher FNR, which specifies that the approach is biased toward majority classes. After employing SMOTE to synthetically balance the minority classes and refining the loss function to penalize the underrepresented classes’ misclassification, all the key measures are enhanced. For plant disease classification, the accuracy is increased from 89.12% to 34.16%, and for pest detection, the MCC is enhanced by 13.8%, representing more balanced and robust datasets. Also, for smart irrigation, the designed model achieves 9.18% improvement in recall, ensuring enhanced reliability in forecasting the irrigation requirements under balanced data conditions. Thus, the results displayed that the implemented approach attained superior predictive performance across all IoT-aided agricultural applications.

**Table 8:** Performance improvement of the designed ISBO-HC-ARMNet before and after class imbalance.

**Terms**	**Before balancing**	**After balancing**	**Improvement**
Class imbalance handling effect on plant disease classification			
Accuracy	89.12	94.16	+5.04
Precision	85.37	94.20	+10.3
Recall	82.45	93.79	+13.8
F1 score	83.88	94.16	+12.3
BA	86.07	94.16	+9.4
FNR	17.55	5.87	−11.68
MCC	0.78	0.92	+17.9
Class imbalance handling effect on pest detection			
Accuracy	90.02	93.78	+4.17
Precision	86.41	93.83	+8.6
Recall	84.92	93.79	+10.5
F1 score	85.64	94.02	+9.8
BA	87.94	93.77	+6.6
FNR	15.08	6.21	−8.87
MCC	0.80	0.91	+13.8
Class imbalance handling effect on smart irrigation			
Accuracy	91.35	95.12	+4.13
Precision	88.09	94.68	+7.47
Recall	86.33	94.25	+9.18
F1 score	87.19	64.46	+8.34
BA	88.56	94.49	+6.69
FNR	13.67	5.75	−7.92
MCC	0.82	0.93	+13.4

### Ablation study

The ablation study of the proposed approach is given in Table 9. Here, the effectiveness of the HC-ARMNet model and the ISBO algorithm is estimated. In the HC vs HC+ARMNet study, combining ARMNet with a hybrid convolution block highly enhanced the accuracy from 88.20% to 91.05%, whereas the incorporation of ISBO further improved to 94.16% and minimized the FNR from 14.40% to 5.87%. The results displayed that the ARMNet and ISBO components improve the feature learning and the parameter tuning effectiveness in the designed work. In the ARMNet vs MobileNet study. The ARMNet outperforms the existing MobileNet in terms of accuracy and FNR. This represents that the designed ARMNet’s recurrent connections and the optimization capability of the ISBO offer highly robust detection performance. Finally, the ISBO vs Adam and SGD study underscores that the designed ISBO surpasses the existing optimizers such as Adam and SGD. These improvements guarantee the ISBO’s superior capability to optimize the parameters and improve the predictive stability. Thus, the ablation study describes that each component, such as hybrid convolution, ARMNet and ISBO, contributes uniquely in enhancing the accuracy, minimizing error rates and attaining a highly reliable and optimized IoT-based smart farming system.

**Table 9:** Ablation study of the proposed work.

Metric	HC Alone	HC + ARMNet	HC + ARMNet + ISBO
HC vs HC+ARMNet			
Accuracy	88.2	91.05	94.16
Precision	87.95	90.92	94.2
Recall	85.6	91.02	93.79
F1-Score	86.76	91	94.16
BA	86.1	90.98	94.16
FNR	14.4	8.98	5.87

ARMNet vs MobileNet			
Metric	MobileNet	ARMNet	ARMNet + ISBO
Accuracy	88.95	90.85	93.78
Precision	88.32	90.75	93.83
Recall	87.1	90.62	93.79
F1-Score	87.71	90.68	94.02
BA	87.5	90.55	93.77
FNR	12.9	9.38	6.21

ISBO vs Adam and SGD			
Metric	SGD	Adam	ISBO
MSE	4.82	3.97	3.21
RMSE	12.23	11.05	10.2
MAE	88.95	77.62	70.72
SMAPE	0.051	0.043	0.0367
BA	89.12	91.04	94.49
F1-Score	87.25	90.34	94.46

### Statistical significance test

Table 10 gives the Friedman Aligned Ranks Test results for the plant disease classification, pest detection, and smart irrigation operations. The results specify that there are no statistically significant differences between the compared heuristic algorithms at a 0.005 significance level. In all analyses, the adjusted p-values exceed 0.5, resulting in the null hypothesis acceptance, which defines that the algorithm works equivalently in terms of media ranks. Overall, the designed model shows promising and effective solutions in the optimization tasks.

**Table 10:** Statistical significance test.

Friedman aligned ranks test – plant disease classification (α = 0.005)			
Comparison	Statistic	Adjusted p-value	Result
CO-HC-ARMNet^30^vs ISBO-HC-ARMNet	2.145	0.621	H0 is accepted
GOA- HC-ARMNet^32^vs CO-HC-ARMNet^30^	1.872	0.811	H0 is accepted
GaOA-HC-ARMNet^31^vs ISBO-HC-ARMNet	1.998	0.732	H0 is accepted
GOA- HC-ARMNet^32^vs GaOA-HC-ARMNet^31^	1.204	0.912	H0 is accepted
CO-HC-ARMNet^30^ vs SBO-HC-ARMNet^26^	1.456	0.845	H0 is accepted
ISBO-HC-ARMNetvs SBO-HC-ARMNet^26^	1.321	0.873	H0 is accepted
GOA- HC-ARMNet^32^vs ISBO-HC-ARMNet	0.932	0.982	H0 is accepted
GaOA-HC-ARMNet^31^vs CO-HC-ARMNet^30^	0.785	0.995	H0 is accepted
GaOA-HC-ARMNet^31^vs SBO-HC-ARMNet^26^	0.653	1	H0 is accepted
GOA- HC-ARMNet^32^vs SBO-HC-ARMNet^26^	0.742	0.998	H0 is accepted

**Friedman aligned ranks test – pest detection (α = 0.005)**			
**Comparison**	**Statistic**	**Adjusted p-value**	**Result**
CO-HC-ARMNet^30^vs ISBO-HC-ARMNet	2.421	0.512	H0 is accepted
GOA- HC-ARMNet^32^vs CO-HC-ARMNet^30^	1.996	0.721	H0 is accepted
GaOA-HC-ARMNet^31^vs ISBO-HC-ARMNet	1.845	0.813	H0 is accepted
GOA- HC-ARMNet^32^vs GaOA-HC-ARMNet^31^	1.276	0.902	H0 is accepted
CO-HC-ARMNet^30^ vs SBO-HC-ARMNet^26^	1.532	0.847	H0 is accepted
ISBO-HC-ARMNet vs SBO-HC-ARMNet^26^	1.412	0.869	H0 is accepted
GOA- HC-ARMNet^32^vs ISBO-HC-ARMNet	0.984	0.971	H0 is accepted
GaOA-HC-ARMNet^31^vs CO-HC-ARMNet^30^	0.864	0.989	H0 is accepted
GaOA-HC-ARMNet^31^vs SBO-HC-ARMNet^26^	0.725	1	H0 is accepted
GOA- HC-ARMNet^32^vs SBO-HC-ARMNet^26^	0.631	1	H0 is accepted

**Friedman aligned ranks test – smart irrigation (α = 0.005)**			
**Comparison**	**Statistic**	**Adjusted p-value**	**Result**
CO-HC-ARMNet^30^vs ISBO-HC-ARMNet	2.305	0.564	H0 is accepted
GOA- HC-ARMNet^32^vs CO-HC-ARMNet^30^	1.987	0.729	H0 is accepted
GaOA-HC-ARMNet^31^vs ISBO-HC-ARMNet	1.763	0.837	H0 is accepted
GOA- HC-ARMNet^32^vs GaOA-HC-ARMNet^31^	1.124	0.918	H0 is accepted
CO-HC-ARMNet^30^ vs SBO-HC-ARMNet^26^	1.398	0.861	H0 is accepted
ISBO-HC-ARMNet vs SBO-HC-ARMNet^26^	1.267	0.883	H0 is accepted
GOA- HC-ARMNet^32^vs ISBO-HC-ARMNet	0.956	0.964	H0 is accepted
GaOA-HC-ARMNet^31^vs CO-HC-ARMNet^30^	0.832	0.991	H0 is accepted
GaOA-HC-ARMNet^31^vs SBO-HC-ARMNet^26^	0.693	1	H0 is accepted
GOA- HC-ARMNet^32^vs SBO-HC-ARMNet^26^	0.721	0.998	H0 is accepted

### Comparative analysis of blockchain vs non-blockchain iot systems

The comparative analysis between the Ethereum blockchain-based IoT smart farming system and the non-blockchain IoT systems is shown in Table 11. This analysis highlights the merits in terms of reliability, transparency and data security of the suggested approach. The blockchain-integrated system highly improves the traceability, data integrity and fault tolerance, attaining effective scores because of its tamper-resistant and decentralized ledger. In addition, the incorporation of smart contracts automates the irrigation and pest control decisions, enhancing the system’s efficiency and minimizing manual intervention. Thus, the Ethereum-based IoT framework guarantees a transparent and secure smart farming system for large-scale agricultural applications.

**Table 11:** Comparative analysis of blockchain vs non-blockchain IoT systems.

Metric/Feature	Non-Blockchain IoT system	Ethereum blockchain IoT system (PoA)	Improvement/Benefit
Data integrity (Score 0–10)	6	10	4
Security (Score 0–10)	6	9	3
Traceability/Auditability (Score 0–10)	5	10	5
Latency (Seconds per transaction)	0.5	5	Moderate trade-off
Transaction throughput (TPS)	200+	~150–200	Slight reduction
Data redundancy/Fault tolerance (Score 0–10)	4	10	6
Automation via smart contracts (Score 0–10)	3	10	7
Energy consumption (Relative units)	1	1.2	Slight increase
Maintenance complexity (Score 0–10)	3	6	Moderate increase

### Scalability analysis

**Fig. 16:**
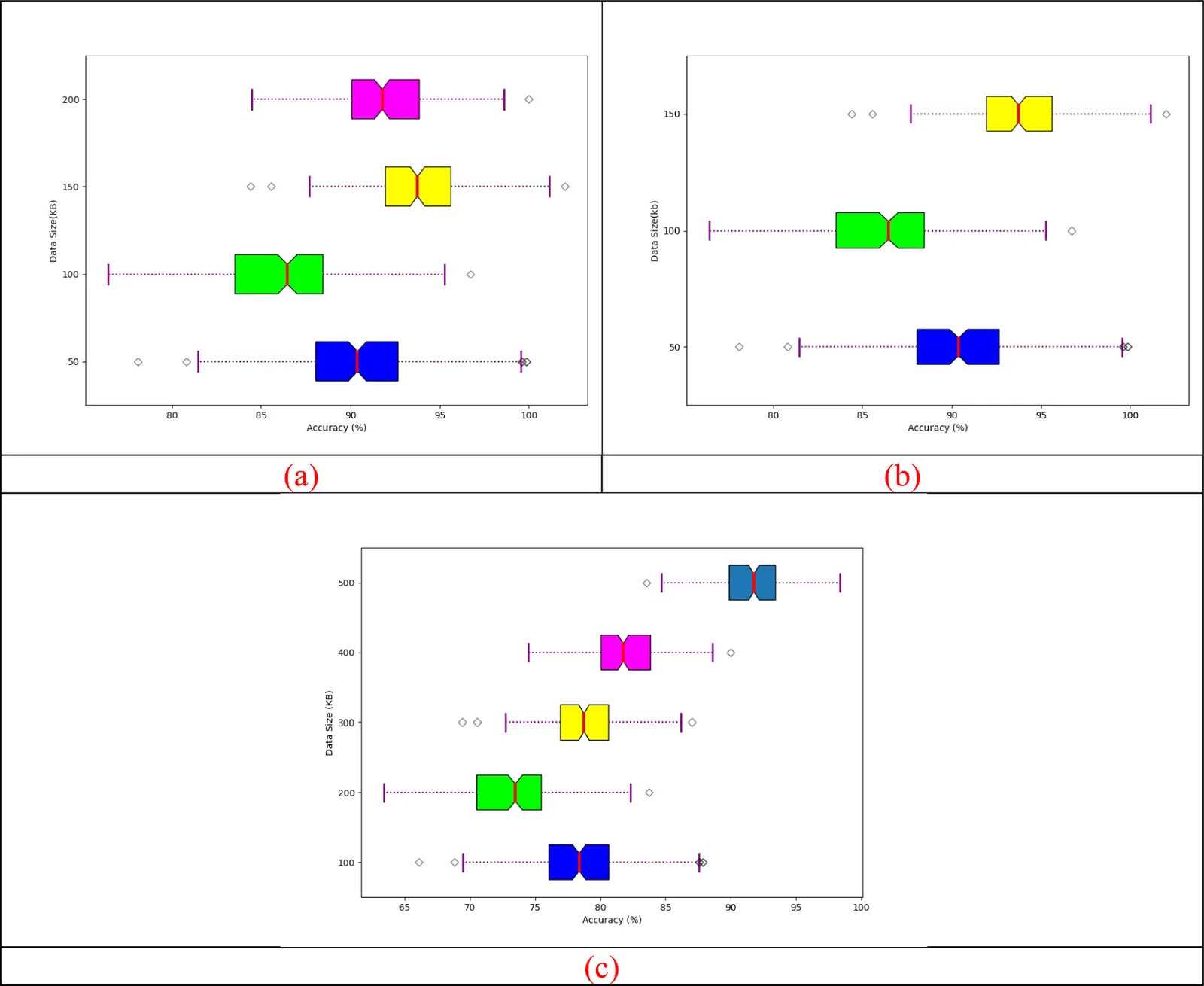
Scalability analysis of the presented approach in terms of “(**a**) Plant disease classification, (**b**) Pest detection and (**c**) Smart irrigation”.

The scalability validation is illustrated in Fig. 16 for the suggested model, which demonstrates its efficiency and robustness as the dataset size increases. By varying the number of samples for plant disease classification, pest detection, and smart irrigation prediction, the model enhances its accuracy. The hybrid 1D/2D convolutions integrated with recurrent layers and MobileNet effectively handle the huge volumes of multimodal IoT data, whereas the class imbalance mitigation models remain efficient across scales. Thus, the designed framework guarantees high scalability, capable of sustaining high predictive performance in large-scale smart farming environments.

## Discussion

The results of the implemented HC-ARMNet + ISBO with Ethereum blockchain approach illustratesignificant advancements in intelligent and secure IoT-based smart farming. Across all three key objectives, such as plant disease classification, pest detection, and smart irrigation prediction, the designed approach achieves high precision, accuracy, and F1 scores. In Section "Convergence investigation of the developed ISBO", the convergence investigation is shown, where the ISBO’s effectiveness is ensured over existing algorithms. In this investigation, the ISBO shows minimized cost function rates, demonstrating that the designed ISBO achieves faster convergence in parameter optimization tasks than the existing algorithms. In Section "Statistical investigation of developed ISBO", the statistical investigation of the ISBO is validated over traditional algorithms. When considering various statistical measures, the implemented ISBO shows promising and superior solutions to the existing algorithms. These validations guarantee that the designed ISBO is relatively suitable for parameter tuning for the HC-ARMNet than the existing algorithms. In Sections "Performance investigation of the plant disease classification process", 6.6 and "Performance investigation of a smart irrigation system", the designed IoT-based smart farming model’s performance analysis is performed, where the ISBO-HC-ARMNet-based plant disease classification, pest detection, and smart irrigation prediction are estimated over existing algorithms and techniques. By considering various positive, negative and error measures, these investigations are performed. From the results, it is elucidated that the designed ISBO-HC-ARMNet outperforms the conventional methods and also achieves high predictive solutions. Further, the overall comparative investigations of the designed ISBO-HC-ARMNet-based plant disease classification, pest detection, and smart irrigation prediction are shown in Sections "Overall comparative investigation of the plant disease classification process", "Overall comparative investigation of the pest detection process" and "Overall comparative investigation of the smart irrigation system". These investigations elucidate that the designed ISBO-HC-ARMNet can contribute to smart farming systems than the existing methods. The comparative investigations of the designed ISBO-HC-ARMNet-based plant disease classification, pest detection, and smart irrigation prediction approaches over the state-of-the-art techniques are presented in Section "Comparative Analysis". These investigations showcase that the ISBO-HC-ARMNet is accurate and effective. Section "Performance Improvement of ISBO-HC-ARMNet Before and After Class Imbalance" presents the performance improvement of the ISBO-HC-ARMNet before and after class imbalance is shown, in which the designed ISBO-HC-ARMNet shows high improvement in terms of accuracy and F1 score. The ablation study in Section "Ablation Study" further ensured the contribution of each component, displaying that integrating hybrid convolutions with the adaptive recurrent MobileNet and optimizing via ISBO highly improved the performance contrasted to baseline approaches and standard optimizers. In addition, the Friedman statistical tests in Section "Statistical Significance Test" indicated that the designed ISBO algorithm is statistically significant, and the comparative blockchain analysis is shown in Section "Comparative Analysis of Blockchain Vs Non-Blockchain IoT Systems", which highly improved the data traceability and integrity via smart contracts. The scalability investigation is presented in Section "Scalability Analysis". All these findings estimate the capacity of the framework to deliver accurate, secure and scalable smart farming solutions, making a highly potential step toward smart, blockchain-powered precision agriculture.

## Conclusion

This work has suggested a new IoT-based smart farming system by utilizing the blockchain mechanism. The soil sensor data and the crop images were aggregated from the IoT sensors in real-time and next integrated this data with the blockchain for conducting the smart irrigation operation. Here, the aggregated data was stored and sent to the Ethereum blockchain with a hash function to protect the data from unwanted activities. The blockchain confirmed the precision and security of the data in the smart farming systems. It enriched the SCM in the farming domain by analyzing the authenticity and employed smart contracts for node authentication. By utilizing the real-time data from the IoT sensors, the HC-ARMNet technique was performed to classify the plant disease and detect the pests in the agricultural farm. Moreover, this technique was used to perform the smart irrigation process. This predictive analytics capacity allowed the farmers to plan for their efficient farming with better ability. To improve the sustainability in agriculture, the parameters in the recurrent MobileNet technique were fine-tuned by the ISBO approach. Finally, the detailed performance analysis was done, and the performance of the implemented process was verified. The precision of the plant disease classification process was 6.91%, 5.31%, 5.31%, and 3.19% higher than the classical algorithms such as CO-HC-ARMNet, GaOA-HC-ARMNet, GOA-HC-ARMNet, and SBO-HC-ARMNet accordingly for the 3rd k-fold value. The accuracy of the implemented pest detection process was 5.55%, 6.66%, 4.44%, and 4.44% improved values than the traditional classifiers such as DNN, 1DCNN, LSTM, and HC-RMNet, correspondingly, for the 200^th^ epoch value. Moreover, the SMAPE of the implemented smart irrigation process was decreased by 73% of CO-HC-ARMNet, 69.2% of GaOA-HC-ARMNet, 46.1% of GOA-HC-ARMNet, and 28.8% of SBO-HC-ARMNet, respectively, for the linear activation function. Hence, it has been proven that the designed IoT-based smart irrigation and pest detection strategies achieved higher accuracy than the other techniques. In future work, the developed IoT-based smart agriculture farming will be strengthened by performing the feature extraction processes with the support of advanced strategies that are lacking in the current work for minimizing the processing time.

**Implications of the research**

The findings of this research work have significant implications for the broader research sector of IoT-based smart farming and precision agriculture. The combination of blockchain technology with innovative deep learning models illustrates an effective pathway toward generating transparent, secure and automated agricultural ecosystems. By integrating hybrid convolutional-recurrent modeling with an optimization-driven learning mechanism, the research develops a new benchmark for managing heterogeneous agricultural data, ranging from time series sensor readings to high-dimensional disease and pest images. The results underscore how resolving the data imbalance, guaranteeing data integrity via blockchain, and allowing edge-efficient computation can collectively improve the decision-making accuracy and reliability in real-world farming environments. Moreover, this work underscores the potential of cross-domain integration, including blockchain, IoT and AI, as a foundation for future research concentrated on autonomous farm management, sustainable agriculture and climate-resilient food production models.

**Limitations of the work**

The designed framework has some limitations, though it is effective in the smart farming system. Initially, the model highly depends on the data quality from IoT sensors. The missing or noisy readings can affect the prediction accuracy. Also, the integration of the components, such as hybrid convolutions, MobileNet, recurrent layers, ISBO and blockchain, adds maintenance overhead and system complexity, highly complicating the large-scale adoption. These problems pave the way for future work, including lightweight model integration and enhanced blockchain efficiency.

## Data Availability

The datasets generated and/or analyzed during the current study are available in the [PlantifyDr Dataset and Pest detection dataset] repository“https://www.kaggle.com/datasets/lavaman151/plantifydr-dataset” and“https://www.kaggle.com/datasets/rtlmhjbn/ip02-dataset”.

## Abbreviations

AI: Artificial intelligence
BA: Balanced accuracy
BM: Book maker
CO: Cuttlefish optimization
DNN: Deep neural network
FDR: False discovery rate
FNR: False negative rate
FPR: False positive rate
GaOA: Gannet optimization algorithm
GOA: Golf optimization algorithm
HC-ARMNet: Hybrid convolution adaptive recurrent MobileNet
IoT: Internet of things
ISBO: Improved secretary bird optimization
LSTM: Long short term memory
MAE: Mean absolute error
MASE: Mean absolute scaled error
MSE: Mean squared error
NP: NPV (Negative Predictive Value)
RF: Random forest
RMSE: Root MEAN SQUARED ERROR
SBO: Secretary bird optimization
SMAPE: Symmetric mean absolute percentage error
SVM: Support vector machine

## Code availability

The implementation code, trained models, and experimental configurations used are publicly available in: “https://github.com/sumanthvmani/-Pest-Detection-and-Smart-Irrigation”.
